# An Atlas of Network Topologies Reveals Design Principles for *Caenorhabditis elegans* Vulval Precursor Cell Fate Patterning

**DOI:** 10.1371/journal.pone.0131397

**Published:** 2015-06-26

**Authors:** Xianfeng Ping, Chao Tang

**Affiliations:** Center for Quantitative Biology and Peking-Tsinghua Center for Life Sciences, Peking University, Beijing, China; East Carolina University, UNITED STATES

## Abstract

The vulval precursor cell (VPC) fate patterning in *Caenorhabditis elegans* is a classic model experimental system for cell fate determination and patterning in development. Despite its apparent simplicity (six neighboring cells arranged in one dimension) and many experimental and computational efforts, the patterning strategy and mechanism remain controversial due to incomplete knowledge of the complex biology. Here, we carry out a comprehensive computational analysis and obtain a reservoir of all possible network topologies that are capable of VPC fate patterning under the simulation of various biological environments and regulatory rules. We identify three patterning strategies: sequential induction, morphogen gradient and lateral antagonism, depending on the features of the signal secreted from the anchor cell. The strategy of lateral antagonism, which has not been reported in previous studies of VPC patterning, employs a mutual inhibition of the 2° cell fate in neighboring cells. Robust topologies are built upon minimal topologies with basic patterning strategies and have more flexible and redundant implementations of modular functions. By simulated mutation, we find that all three strategies can reproduce experimental error patterns of mutants. We show that the topology derived by mapping currently known biochemical pathways to our model matches one of our identified functional topologies. Furthermore, our robustness analysis predicts a possible missing link related to the lateral antagonism strategy. Overall, we provide a theoretical atlas of all possible functional networks in varying environments, which may guide novel discoveries of the biological interactions in vulval development of *Caenorhabditis elegans* and related species.

## Introduction

It has been suggested that general design principles underlie networks that can robustly achieve a particular biological function [1–4]. The robustness of biological functions, i.e. the capability of reliably executing a function in spite of complex environments and genetic perturbations, is believed to impose constraints on the evolutionary process of the underlying networks to perform those functions. For example, recent studies have identified the design principles for the topologies that perform biological functions in various systems, such as *Drosophila* segment polarity [5], biochemical adaptation [6,7], biological oscillators [8] and the bistability underlying mammalian cell-cycle entry [9]. Ignoring the details of molecular implementation, these principles help to simplify and demystify the complexity that may otherwise be difficult to comprehend. However, extracting general design principles for other important biological systems, such as cell fate patterning in the differentiation of the VPCs in *C*. *elegans*, remains a challenging task [10], which is essential to clarify from two aspects: (i) further understanding the relationship between function and topology; and (ii) providing new insights into the underlying networks that only partial knowledge exists on the molecular mechanisms and pathways.

**Fig 1:**
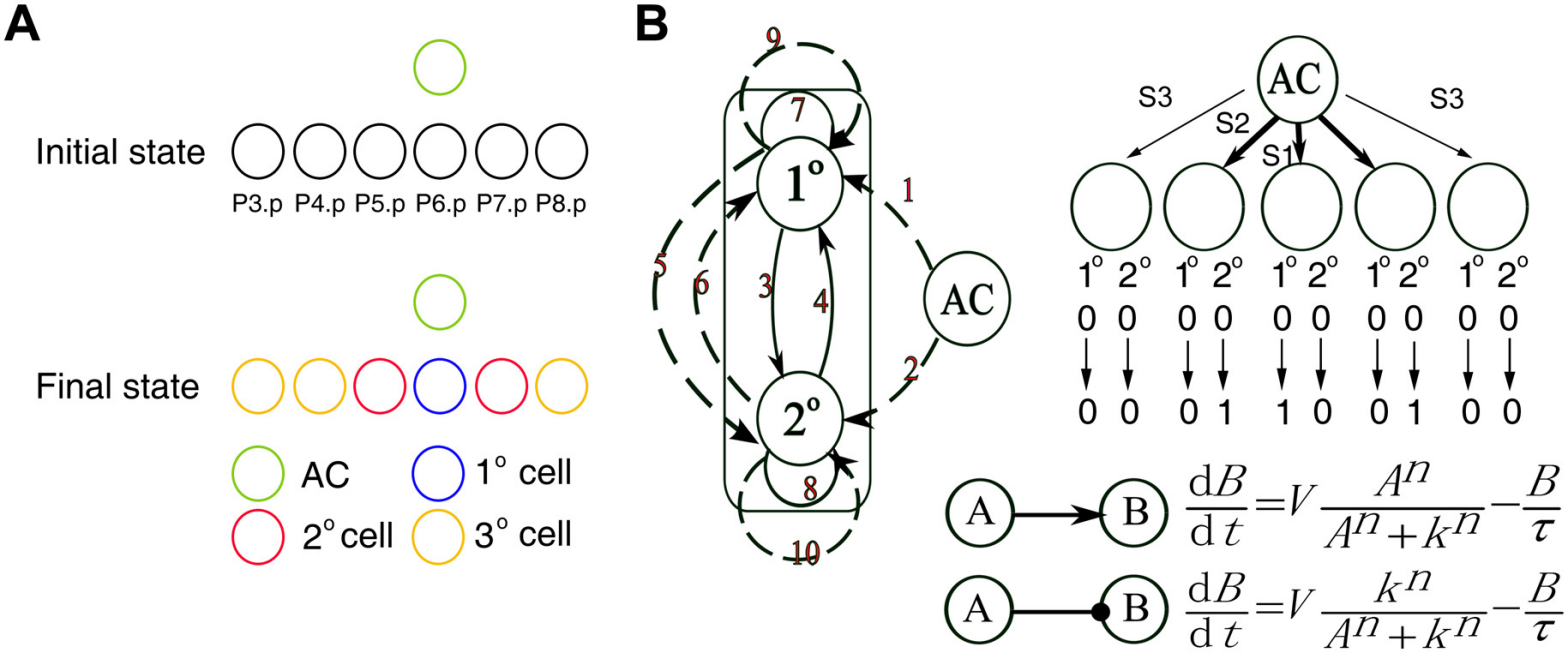
The *C*. *elegans* VPC patterning system and the coarse-grained model. (A) VPC differentiation. (B) Coarse-grained modeling of VPC patterning. The two-node model with 10 links numbered from 1 to 10 is shown in the left panel. Dashed lines represent intercellular interactions and solid lines intracellular interactions. The modeling system of five Pn.p cells along with their initial and target values is shown in the upper-right panel. The ODE functions of two examples are shown in the lower-right panel.

Vulval development in *C*. *elegans* has served as a paradigm for cell fate determination [10,11]. The nematode’s vulva is formed from the descendants of six multipotent VPCs, named P3.p to P8.p, that adopt one of three cell fates (Fig 1A). Early evidence showed that their fates depend on their distances to an anchor cell (AC), which is near the VPCs and secretes an epidermal growth factor (EGF) signal [11,12]. The VPC closest to the AC (P6.p) becomes a primary (1°) cell; the VPCs at an intermediate distance (P5.p and P7.p) become secondary (2°) cells; and the more distant ones (P3.p, P4.p and P8.p) become tertiary (3°) cells (Fig 1A). Two important pathways have been uncovered that contribute to the VPC patterning: the inductive signal pathway and the lateral signal pathway [11,13–16]. More specifically, the inductive signal is transduced by the receptor-tyrosine kinase (RTK) pathway with diffusible EGF from the AC as its ligand. The lateral signal is transduced by the Notch pathway between the VPCs themselves, with Notch as its receptor. The ligands of the Notch pathway have two forms: diffusible DSL-1 and membrane-bound LAG-2 and APX-1 [17]. Evidence showed that the two pathways and their crosstalk induce VPC patterning [18].

Two general models have been proposed to quantify the process of VPC patterning: the morphogen-based and the sequential induction models. In the morphogen-based induction model, EGF forms a gradient that triggers different levels of RTK pathway activity and thereby distinct Pn.p fates as a function of distance to the AC [12]. In the sequential model, the fates of the P5.p and P7.p cells are triggered by a lateral signal, rather than an inductive signal [19]. With different strategies to induce the fate of the 2° cells, the two models seem to be unrelated to each other, though both have experimental support [20]. Recent efforts were devoted to reconciling the two seemingly conflicting models either by computational modeling [21] or searching for novel biological mechanisms experimentally [22,23]. Various aspects of vulval development have been investigated, such as the intracellular trafficking of signaling molecules and the mutation studies of VPC patterning system [24,25]. However, all of the previous computational studies on VPC patterning were based on specific networks, which were inferred from experiments and may be incomplete. Here, we used a distinct theoretical approach to explore the design principles of VPC patterning. Instead of focusing on a single experimentally-derived network, we used an exhaustive computational search to identify the space of networks that can robustly perform the function of vulval precursor cell fate patterning.

We obtained an atlas of all the possible robust functional topologies under different conditions of levels of source signal from the AC, various proportions of diffusible and transmembrane intercellular regulation, and two different regulatory logical rules. We identified three potential strategies that the system can use to achieve VPC patterning. Our analysis revealed the design principles of all the topologies that are capable of VPC patterning and provided a resource to guide novel discoveries in *C*. *elegans* vulval development.

## Results

### Coarse-graining the biological network and constructing a model

We started from a functional point of view to ask, given the geometry of the system and a source of a graded ‘long range’ signal, what kind of network topology robustly stands out for the patterning task? Here, we used a coarse-grained modular model, in which all genes and proteins expressed/involved for 1° cell fate are represented by one node (1° Node), and those for the 2° cell fate by another (2° Node). For simplicity, the 3° cell fate is represented as neither the 1° nor 2° cell fates (i.e. both nodes are zero in the cell). Thus each cell has two nodes, and depending on which node wins, has the potential of being either of the two fates, or being the 3° fate if none of the nodes is induced. This reduction is desirable because it enables us to perform a much more comprehensive and theoretical analysis, while it does not significantly affect the core properties of the system.

We then proceeded to enumerate topologies of networks with two nodes in each cell and the AC along with intra- and intercellular interactions. Each node can be regulated by the AC intercellularly, as well as by itself and the other node (both intracellularly and intercellularly), resulting in 10 directed links, numbered from 1 to 10 (defined as link number) (Fig 1B). (Note that for simplicity the topology is drawn for one cell while the intra- and intercellular interactions are the same for all Pn.p cells, except for the interactions from the AC that act on three cells as indicated in Fig 1B). Each link has three possibilities: positive regulation (“P”), negative regulation (“N”), or absent. This results in a library of 3^10^ = 59, 049 topologies that encompasses all possible link combinations. We used the link number and the property of a link to represent it in a typical topology. For example, “1P” represents the #1 link with positive regulation. Then each topology was named as a combination of links, such as “1P-5P”.

To investigate whether and how the functional topologies change in response to a range of source signal from the AC, we varied the range of the source signal by fixing the strength of the source input to the 1° cell (S1 = 1) and varying its strength to the 2° cells (0 ≤ S2 <1) (Fig 1B). Because the 3° cells receive weak source signal, in most simulation, the strength of AC signal to the 3° cells (S3) is set as 0 unless specified. We considered two types of intercellular interactions among Pn.p cells, diffusible and membrane-bound (e.g. DSL-1 and LAG-2 in the Notch pathway), and randomly set a ratio of the two types for each circuit. We used a model of ordinary differential equations (ODEs) to quantitatively assess the ability of each topology to perform the patterning function. We note that for multiple regulatory links on one node, different logic rules may result in varied dynamics of the same topology. We used the “AND” rule and “Combined AND and Additive” rule in our simulation. In the “AND” rule, all regulatory links on a node, positive and negative, adopt the “AND” logic (multiplicative); while in the “Combined AND and Additive” rule, positive regulations adopt the “OR” logic (additive) and negative regulations take a more dominant role by adopting the “AND” logic. We generated a space of 10,000 parameter sets, which were randomly sampled with values within the biologically-feasible ranges. This parameter space serves as a surrogate of the space of varied molecular implementations and environmental conditions. To measure the robustness of the topologies, we used the quantity *Q = m/n*, where *n* is the number of random parameter sets used in the sampling, and *m* is the number of those sets that can perform the function.

### Searching for functional topologies

**Fig 2:**
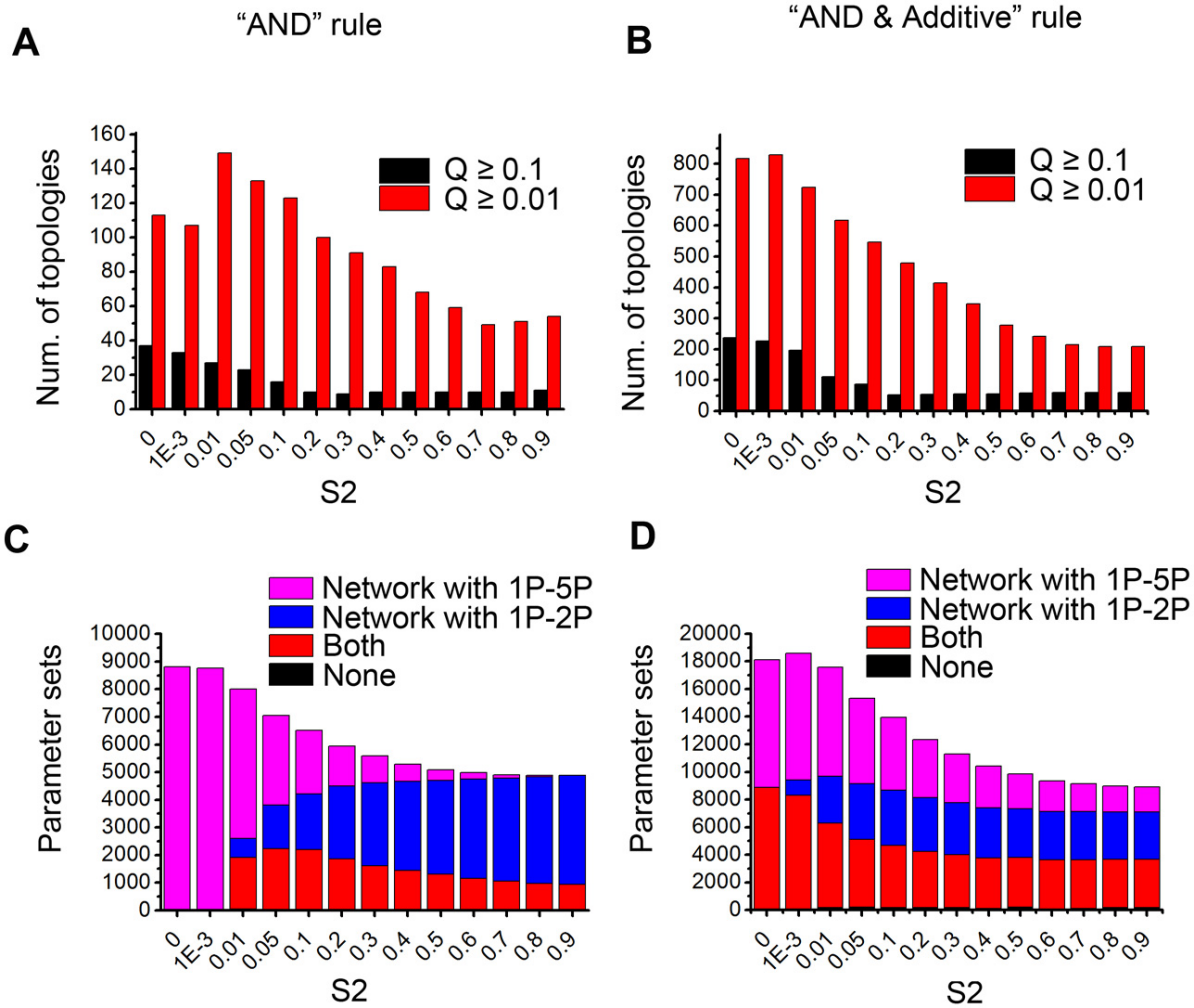
Summary of the topologies and parameter sets that achieve the patterning function. (A, B) Number of topologies with *Q* ≥ 0.1 and *Q* ≥ 0.01 for different S2 values with “AND” rule (A) and “Combined AND & Additive” rule (B). (C, D) For different S2 values, the distribution of parameter sets for functional topologies that contain module 1P-5P or 1P-2P with “AND” rule (C) and “Combined AND & Additive” rule (D). In the legend of C and D, “1P-5P”, “1P-2P”, “Both”, and “None” indicate the parameter sets on which there are only topologies with 1P-5P, only topologies with 1P-2P, both topologies with 1P-5P and topologies with 1P-2P, and none of the topologies with 1P-5P or 1P-2P.

We searched the topologies of networks that robustly achieve VPC patterning for different values of the signal input to the 2° cells, while fixing the source input to the 1° cell (see Materials and Methods). S1 and S2 Tables list all robust topologies (*Q* ≥ 0.1) for different S2 with “AND” and “Combined AND and Additive” rules, respectively. Only a small proportion of topologies have *Q* ≥ 0.1 or *Q* ≥ 0.01 (Fig 2A and 2B). For example, for S2 = 0 and with the “AND” rule, there are only 37 topologies (from a total of 59,049) with *Q* >0.1 and 113 topologies with *Q* >0.01. This result agrees with previous studies in other systems [5,6]: function constrains the topologies of networks.

Then we checked the parameter space of the total 10,000 parameter sets and asked: for each parameter set, are there any networks with that parameter set that can perform the patterning function? For the “AND” rule with S2 = 0, more than 8,000 parameter sets have at least one network that can achieve the function, the number decreases as S2 increases to 0.1, and then becomes steady after S2 gets larger (Fig 2C). This analysis suggests that a long-range source signal is not advantageous to pattern formation. Furthermore, we found that most of the topologies that perform the patterning task with at least 1 parameter set contain at least one of the two motifs: 1P-5P, which agrees with the sequential induction model, and 1P-2P, which is consistent with the morphogen-based model. When S2 is small, the proportion of parameter sets that use the motif 1P-5P is larger than that using 1P-2P. In contrast, the proportion of those using 1P-2P prevails when S2 becomes larger. Analysis using the “Combined AND and Additive” rule results in similar conclusions (Fig 2D): 1P-5P plays dominant role in lower S2 while 1P-2P prevails in higher S2.

The two different logic rules do lead to some different results. For the “AND” rule, the two motifs 1P-5P and 1P-2P are mutually exclusive in most cases, i.e. most robust functional topologies contain only one of the two motifs. While for the “Combined AND and Additive” rule, the motif 1P-2P-5P, which can be considered as the combination of the two motifs 1P-5P and 1P-2P, is seen in many functional topologies. For the “AND” rule, the investigation in the space of all the functional topologies reveals that most topologies can be stratified into two classes: the first contains 1P-5P and can be functional in lower S2, and the second has 1P-2P and can be functional in higher S2 (S1 Fig). It suggests that there are different strategies to achieve the functions and these depend on the signal strengths of S2. It is of note that the modules 1P-5P and 1P-2P indicate different ways to induce the 2° fate. On the other hand, the additive rule for positive regulations in the “Combined AND and Additive” logic makes it possible for a topology to permit the existence of both ways to induce the 2° fate, even though only one of the ways plays the essential role.

### Minimal topologies reveal VPC patterning strategies

**Fig 3:**
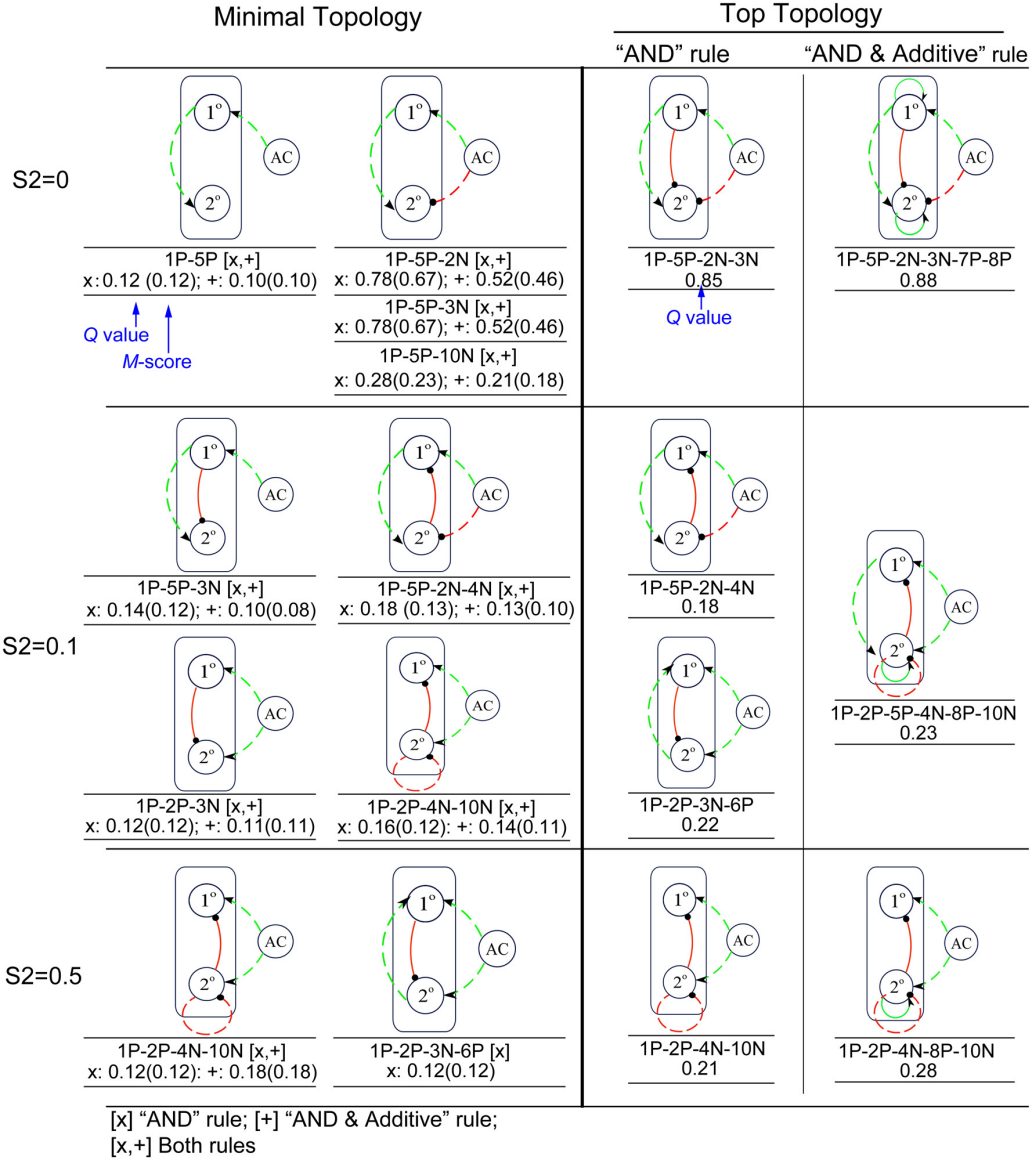
Minimal topologies and top topologies for S2 = 0, 0.1, and 0.5. The left column lists all the identified minimal topologies with both regulatory rules. “AND” rule is marked as “x”, and “Combined AND & Additive” rule is marked as “+”. For each rule, the *Q* value and *M*-score are labeled below the topology, where *M*-score is labeled in parentheses directly following the *Q* value. Most minimal topologies are shared by both regulatory rules except 1P-2P-3N-6P for S2 = 0.5, which is minimal topology only with “AND” rule. The top topologies identified with “AND” rule (middle column) and “Combined AND & Additive” rule (right column) are shown. Typical topologies with highest *Q* values are selected. *Q* value for each topology is labeled below the topology.

To investigate how the uncovered topologies achieve pattern formation, we first analyzed minimal topologies, which represent the skeletons that are capable to perform the VPC patterning. We define minimal topologies as follows: If two distinct topologies, A and B, both perform functions under a given parameter set P, and the architecture of A contains all the links of B, then B is said to be smaller than A. If among all the topologies, there are none smaller than B, then B is said to be minimal for the parameter set P, and this means that all the links of B are essential to achieve the function for the given parameter set. For each topology, we calculated the *M*-score as the proportion of parameters for which the topology is minimal. We define a topology as a minimal topology if it has a *M*-score ≥ 0.1. By definition, minimal topology is the simplest (as few links as possible) and robust (*Q* ≥ 0.1) topology and the *M*-score measures the reduced *Q* value if any links are removed. As expected, these identified minimal topologies fall into two types: including the motif either 1P-5P or 1P-2P (Fig 3). The minimal topologies with 1P-5P prevail for smaller S2, and those with 1P-2P for larger S2.

We searched for minimal topologies with both “AND” and “Combined AND and Additive” rules. Fig 3 lists all the identified minimal topologies for the low (S2 = 0), medium (S2 = 0.1) and high (S2 = 0.5) S2 with both rules. We found that the two rules share most of the minimal topologies, indicating that the two rules result in the similar core strategies for the topologies to achieve the patterning function. All the minimal topologies contain the motif either 1P-5P (for low and medium S2) or 1P-2P (for medium and high S2) exclusively, indicating distinct strategies to achieve VPC patterning.

**Fig 4:**
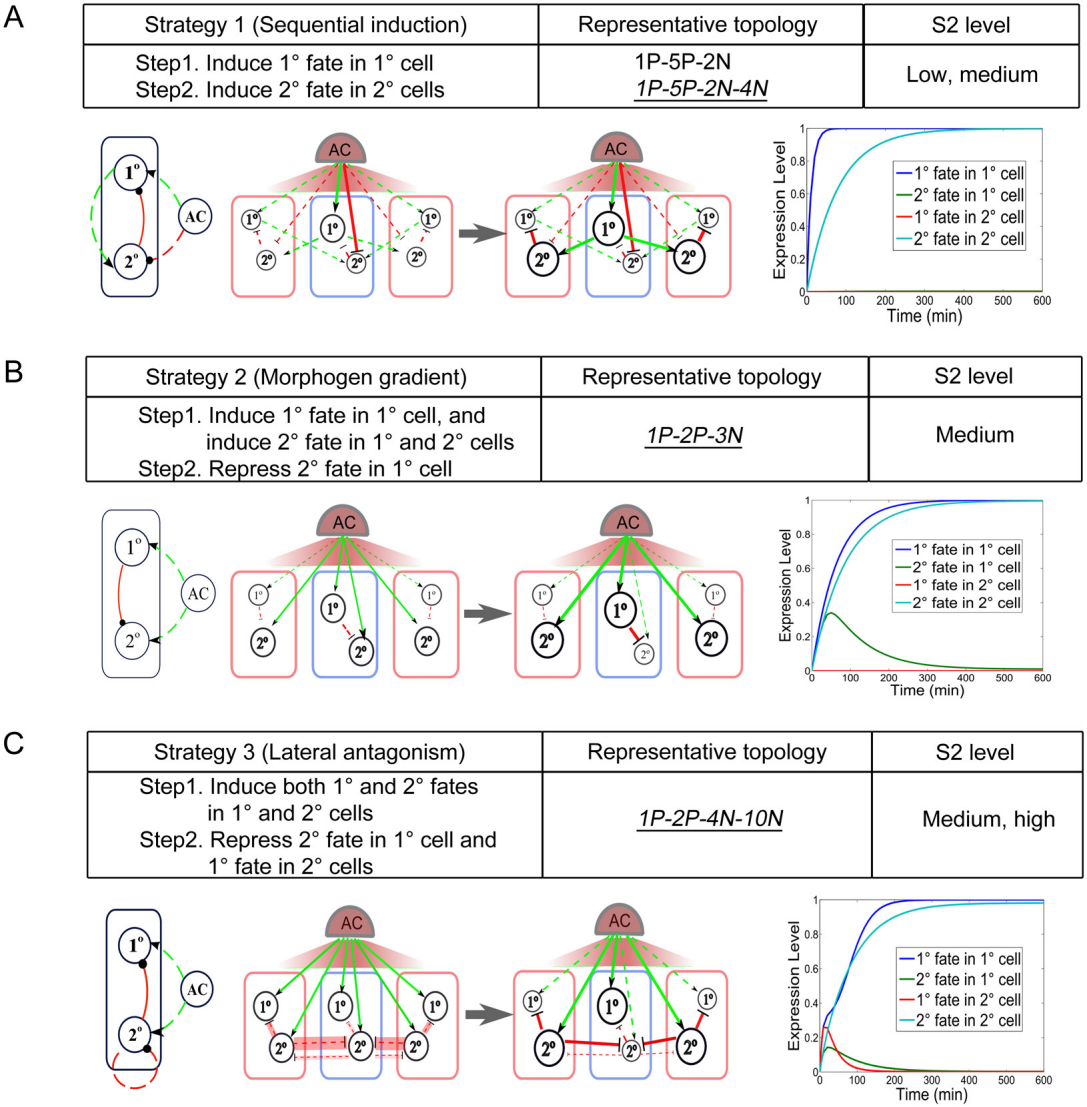
Different strategies to achieve the pattern. Sequential induction strategy (A), morphogen gradient strategy (B), and lateral antagonism strategy (C) are shown. These strategies are common with both “AND” and “Combined AND & Additive” rules. For each strategy, a simple description of the strategy, representative topology, and the S2 level are listed in the table. Below the table shows the mechanism of representative topology: on the left is a sketch of the topology; in the middle is the graph that shows the regulation among the AC, 1°, and 2° nodes in the 1° (middle) and 2° cells (two sides), where the heavy full lines indicate acting or strong regulation and fine dashed lines indicate no or weak regulation; on the right draws the dynamical value of each node in the 1° cell and 2° cells with increasing time.

In order to investigate how these minimal topologies achieve the function, for each topology we looked into the dynamics of each fate in each cell during the simulation. All of the minimal topologies are found to follow the three strategies illustrated in Fig 4 and described below. The first strategy is adopted for low to medium S2 and employs sequential induction with the motif 1P-5P, in which AC induces 1° fate in its nearest 1° cell which in turn induces 2° fate in the two neighboring 2° cells. Negative links function to repress the 2° fate in 1° cell or 1° fate in 2° cells if necessary, such as 1P-5P-2N-4N (Fig 4A). The second strategy, which is employed by 1P-2P-3N, agrees with the morphogen gradient model, in which 1° and 2° cells read the AC signal directly and make their fate choices according to the signal strength they receive, and only functions at the medium S2 levels (Fig 4B). At first, both 1° and 2° fates are induced in the 1° cell, but only 2° fate is induced in the 2° cells, which is ensured by special constraints of parameter sets (see S1 Text). Then the activated 1° fate further represses 2° fate in the 1° cell. The third strategy, employed by 1P-2P-4N-10N for medium to high S2 levels, works as follows (Fig 4C): first, both 1° and 2° fates are induced in both 1° and 2° cells, then the 2° fate in 1° and 2° cells competes to inhibit each other (10N). In this competition, the 2° fate in the 2° cells wins, and the 2° fate in the 1° cell is inhibited. Consequently, 1° fate in the 2° cells is also inhibited (4N). This strategy, which we name it as “lateral antagonism”, is distinct from the morphogen gradient strategy described above, although they both contain module 1P-2P. The lateral antagonism strategy mainly depends on the competition or mutual inhibition of 2° fate in neighboring cells, while the morphogen gradient strategy mainly depends on the graded signaling from the source cell. We also found a strategy only used for “AND” rule by 1P-2P-3N-6P. In this topology, there are two positive regulatory links on the 1° fate, from AC and the 2° fate of the neighboring cells, which cause different behaviors between the two rules. For the “AND” rule, the activation of the 1° fate needs not only positive regulation from the AC (1P) but also the intercellular positive regulation from the 2° fate (6P), which makes this strategy available with the “AND” rule, exclusively (S2 Fig). In this strategy, 2° fate is induced first in both 1° and 2° cells, then 1° fate is only induced in the 1° cell, and then 2° fate in the 1° cell is repressed.

### Design principles of top topologies

Next we searched the top topologies that are most robust at different S2 levels (Fig 3 and S3 and S4 Tables). The clustering of the top topologies shows that for both “AND” and “Combined AND and Additive” rules, there are two main clusters: sequential induction (1P-5P) based topologies with high *Q* values for lower S2, and morphogen gradient and lateral antagonism based (1P-2P) topologies with high *Q* values for higher S2 (S3 Fig).

Top topologies are built upon minimal topologies and usually are the combinations of minimal topologies with either the same strategy or distinct strategies (Fig 3 and S3 and S4 Tables). For the “AND” rule, the topologies that contain 1P-5P with the combination of 2N, 3N, or 4N often rank as top topologies and use sequential induction strategy at low and medium S2 levels, while the minimal topology 1P-2P-4N-10N, 1P-2P-3N-6P and the combination of them rank as top topologies at medium and high S2 levels and use the corresponding strategies described above. For the “Combined AND and Additive” rule, similar results were found: top topologies are built upon 1P-5P at low and medium S2 levels, and upon 1P-2P-4N-10N at medium and high S2 levels. In addition, adding self-activation loops (7P, 8P) often increases the *Q* values. Notably, for each level of S2, we found topologies with 1P-2P-5P, which means the combination of different strategies, ranked as top topologies. However, only one strategy plays the dominant role at specific S2 level. At the low S2 levels, 2P is not an essential link—the minimal topologies only use motif 1P-5P. For example, both 1P-2P-5P-3N-7P-8P and 1P-5P-3N-7P-8P have a *Q* value of 0.84 for S2 = 0, indicating that 2P is a neutral link. These topologies mainly use sequential induction strategy. In contrast, for the medium and high S2, most top topologies with 1P-2P-5P-4N-10N mainly use lateral antagonism strategy, and 5P is not essential.

**Fig 5:**
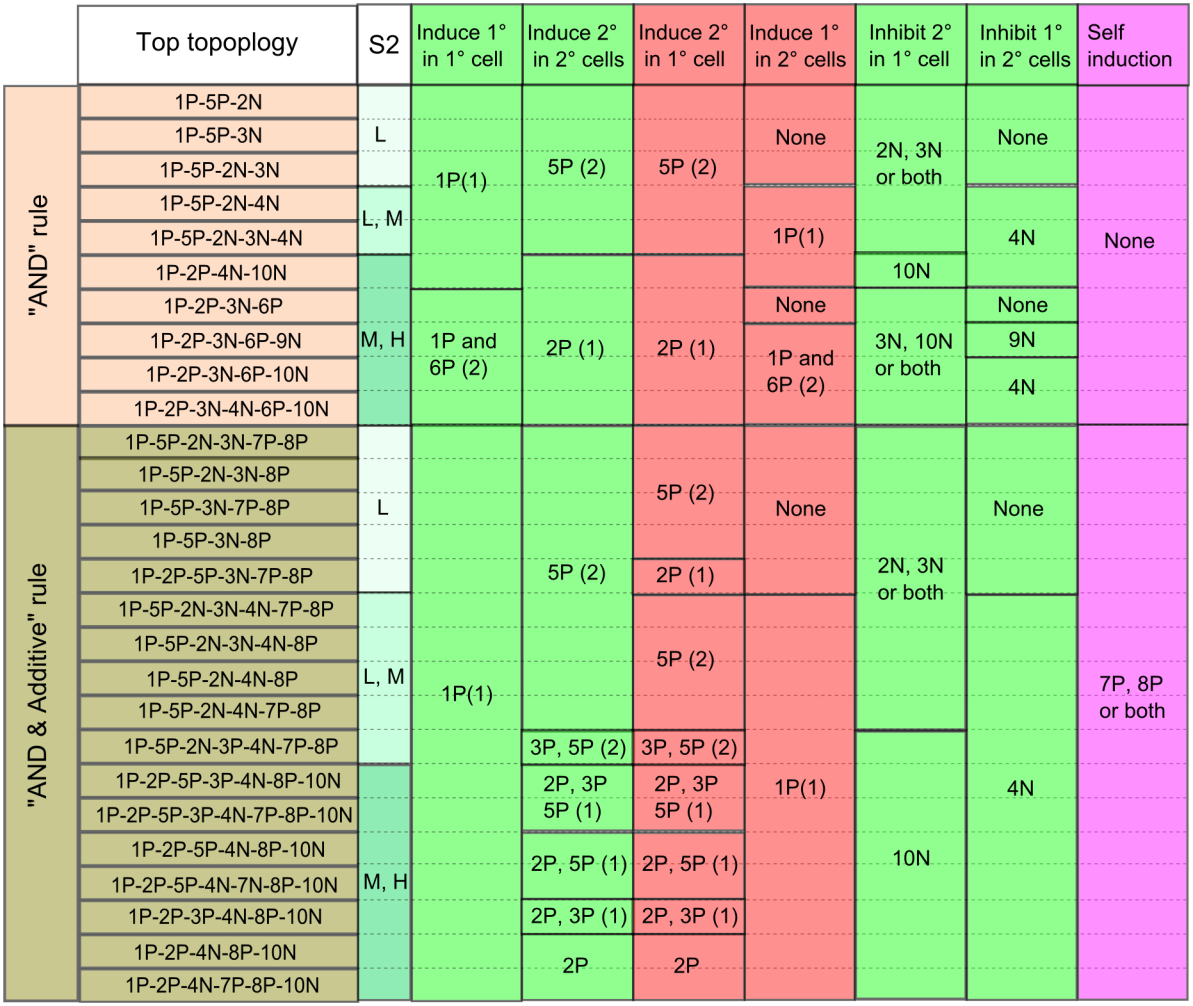
A map of links for each top topology to the decomposed functions for VPC patterning. (Left columns) Top topologies ranking within the top 5 for each S2 are listed. Both cases of the “AND” rule and the “AND & Additive” rule are included. (Middle column) The S2 column shows the S2 levels at which the topologies performed the patterning robustly. L, low level; M, medium level; H, high level. (Right columns) For each topology (each row), its links are listed in different columns according to their functions. It should be noted that among these functions, inducing 2° fate in 1° cell and 1° fate in 2° cell (in red color) have adverse effects on the topologies’ performance of patterning. The numbers in parentheses indicate the sequential order of the functions for positive regulation. The same number means the functions have no causal or sequential relationship.

Although top topologies share some common patterning strategies, they employ different links to implement the strategies. Fig 5 shows how different top topologies employ distinct links to implement modular functions that contribute together to the patterning. Except for the links 7P and 8P that mean positive regulation by nodes 1° and 2° themselves, all links can be assigned one or two of the following six functions: inducing the 1° fate in the 1° cell, inducing the 2° fate in the 2° cells, inducing the 1° fate in the 2° cells, inducing the 2° fate in the 1° cell, inhibiting the 1° fate in the 2° cells, inhibiting the 2° fate in the 1° cell. With distinct implementation of each component function, the integrated VPC patterning program can be achieved by various combinations of different links.

Given the diversity and redundancy of the topological implementations of VPC patterning, top topologies also show high structural flexibility. In this study, we define the neighbors of a given topology by changing the properties (positive, negative, or absent) of one of its links, and the structural flexibility of a given topology is estimated by the average robustness of its topological neighbors that perform the same patterning function [9]. We found that for both the “AND” rule and the “Combined AND and Additive” rule, there are tight correlations between structural flexibility and functional robustness (S4 Fig), especially for those with the “Combined AND and Additive” rule, suggesting the capability of the identified top topologies to remain functional in despite of alterations of their links.

### Diffusible *versus* membrane-bound intercellular regulation

**Fig 6:**
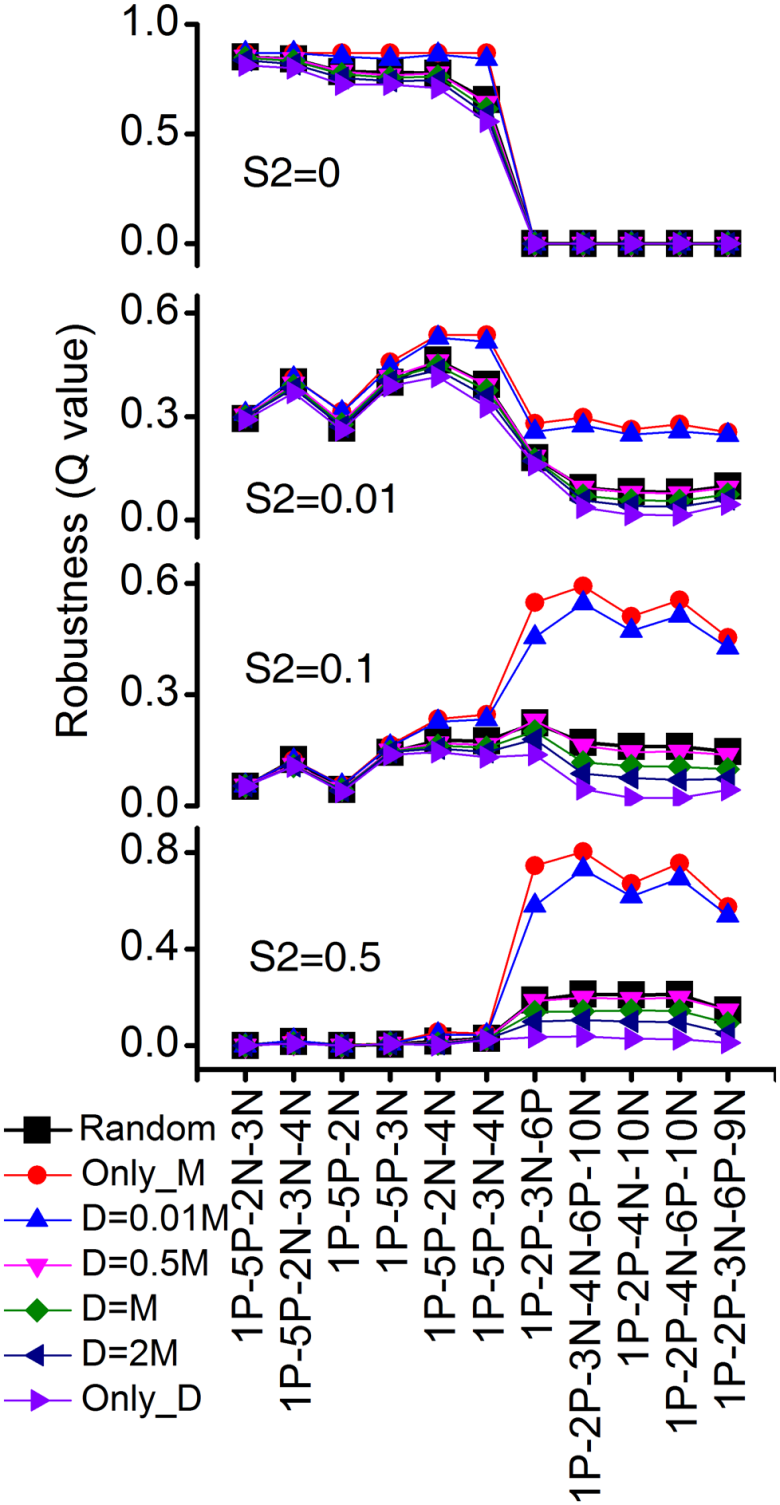
Top topologies for different ratios of diffusible to membrane-bound intercellular regulation. *Q* values of top topologies for different ratios of diffusible to membrane-bound intercellular regulation are plotted. “Only_M” means only membrane-bound and “Only_D” means only diffusible. “Random” means the ratio is evenly sampled from (0, 1). “D = 0.01M, 0.5M, M, 2M” means the ratios of diffusible to membrane-bound are 0.01, 0.5, 1, and 2, respectively. Four cases of different S2 values (0, 0.01, 0.1 and 0.5) are shown from top panel to bottom panel.

As both membrane-bound (LAG-2) and diffusible (DSL) signals are found in the intercellular signaling among fate-patterning cells and the two signal-induced regulations act differently in the system (the membrane-bound signal induced regulation acts only on the adhesive cells, but the diffusible signal induced regulation acts not only on these cells but also on the source cell itself) [17], we further asked how different proportions of the two kinds of intercellular regulation affect the robustness of the uncovered topologies. Instead of using a random proportion of the two kinds of regulation, we repeatedly enumerated all the coarse-grained networks by fixing the proportion and estimated the robustness of each topology from 10,000 samples of parameter sets. For simplicity, we used “AND” rule for this simulation. Comparing the robustness of the previously discovered top topologies for different proportions of the two kinds of regulation, we found that with a higher proportion of membrane-bound intercellular signaling, the uncovered topologies, both types of topologies with lower and higher S2 respectively, have higher *Q* values than those with a lower proportion of membrane-bound regulation (see Fig 6 and S5–S8 Tables). The advantage of membrane-bound regulation for functional robustness is considerable for topologies with 1P-2P for larger S2 values. For example, for S2 = 0.5, the *Q* value of the topology 1P-2P-3N-4N-6P-10N is 0.80 for membrane-bound regulation only, and 0.04 for diffusible regulation only. This suggests that high robustness of topologies for high S2 requires a very high proportion of membrane-bound ligands. Though the advantages are not as evident as those for topologies with 1P-2P, larger proportion of membrane-bound regulation also facilitates some networks with 1P-5P to become more robust, such as 1P-5P-3N-4N for S2 = 0.01 (*Q* = 0.54 for membrane-bound regulation only and *Q* = 0.34 for diffusible regulation only). However, for an extremely low S2 value, such as S2 = 0, the most robust topologies, such as 1P-5P-2N-3N-4N, perform invariantly for varying proportions of the two kinds of intercellular regulation.

### Behaviors of topologies under the simulated mutant conditions

**Fig 7:**
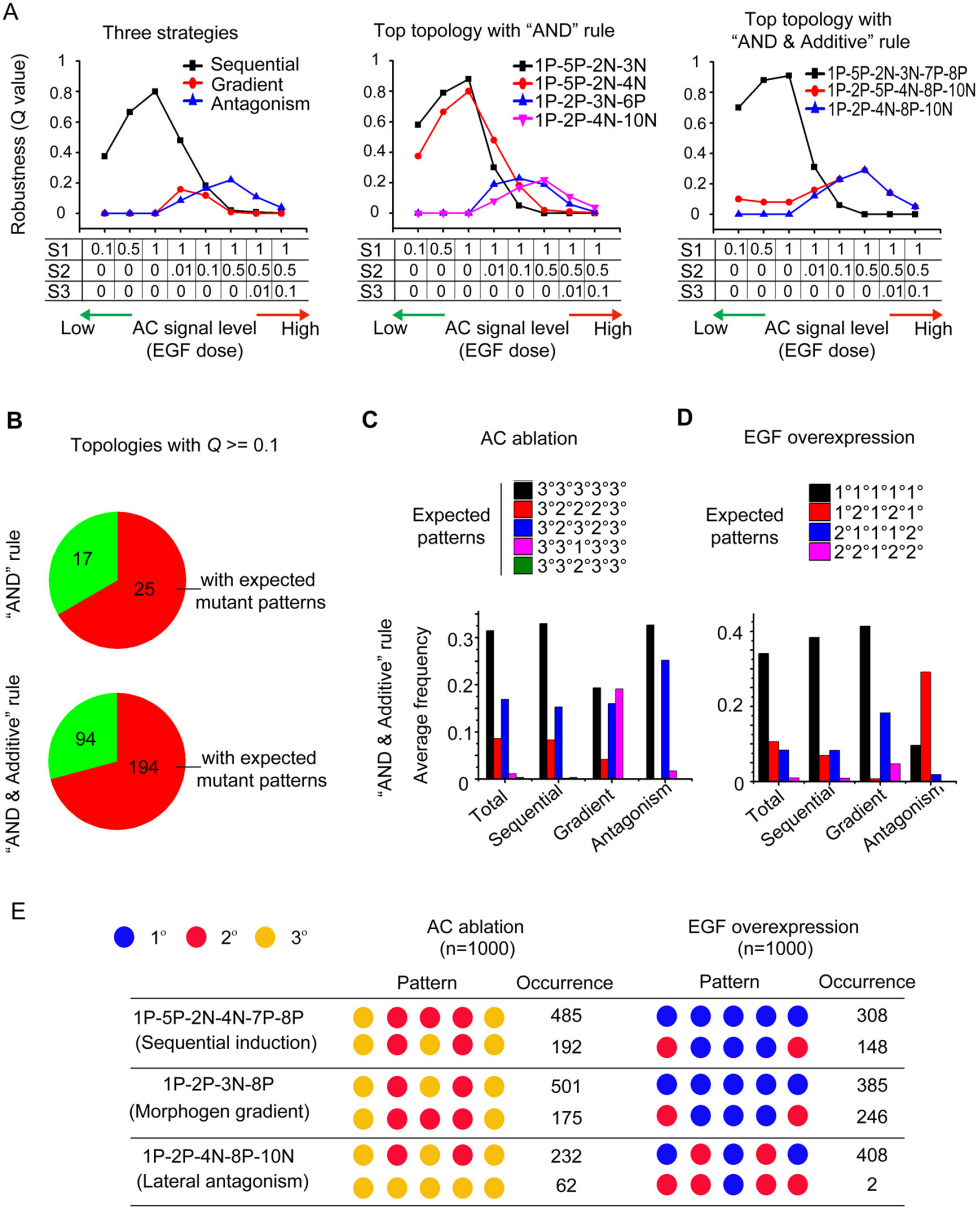
Behaviors of topologies under simulated mutant conditions of AC signaling. (A) Robustness (*Q* value) of topologies under varying AC signal conditions; *left*: representative topologies for three strategies, from Fig 4; *middle*: top topologies with “AND” rule, from Fig 3; *right*: top topologies with “AND & Additive” rule, from Fig 3. (B) Pie charts show the number and percentage of robust topologies that reproduce expected mutant patterns reported in Ref. [26] with “AND” rule (*top*) and “Combined AND & Additive” rule (*bottom*). (C, D) Average frequency of expected mutant patterns produced by different types of topologies in 1,000 runs of simulation with functional parameters under AC ablation (C) and EGF overexpression (D). The expected mutant patterns are from Ref. [26]. Results of simulation in “Combined AND & Additive” rule are shown. (E) Expected mutant patterns reproduced by representative topologies for three strategies under AC ablation and EGF overexpression. The top two most-frequent patterns along with corresponding occurrences from total 1,000 runs of simulation are shown. Simulation was modeled with “Combined AND & Additive” rule.

Previous experiments have shown that perturbations of LIN-3/EGF signaling or Notch signaling may result in error cell fate patterns in VPC system [26,27]. Herein we sought to investigate how identified robust topologies perform under the variations of source signaling and lateral signaling in our model. We first altered the AC signal strength and see how robustness changes. We simulated the AC signal level from low to high by varying S1, S2, and S3 from 0 to 1, while keeping S1 > S2 > S3 (Fig 7A). As expected, from low to high AC signal, all three strategies and the identified top topologies show a similar pattern of change in robustness: first increases and then decreases, with the peak robustness at different places (low AC signal for sequential induction, medium for morphogen gradient induction and high for lateral antagonism strategy) (Fig 7A, S9 and S10 Tables). We also investigated the *Q* values of topologies with three strategies under the conditions of varying S1 and S2 concordantly (S5 Fig). Results show that increasing both S1 and S2 concordantly leads to similar results as obtained by fixing S1 = 1 and only increasing S2 level (S5A Fig). On the other hand, slightly decreasing S1 and S2 concordantly to a half (0.5×) does not change *Q* values much, while decreasing S1 and S2 to one tenth (0.1×) decreases the *Q* values (S5B Fig), which is consistent with the results under the conditions of decreasing AC signal above (Fig 7A).

We next investigated what cell fate patterns the identified topologies with functional parameters would result in under the simulated mutant conditions of AC ablation and LIN-3/EGF overexpression, and compared to the experimental results [26]. In the work by Félix [26], several error cell fate patterns were observed under the mutation of AC signaling in *C*. *elegans* and other *Caenorhabditis* species. We asked whether our topologies could reproduce the observed patterns in our simulation. We executed mutant simulation for all the robust topologies (*Q* ≥ 0.1 for at least one signal condition, S1 and S2 Tables). Simulation results show about 2/3 of robust topologies are able to reproduce expected error patterns observed in previous experiments (Fig 7B, S11 and S12 Tables). For all the topologies that reproduce expected error patterns, on average, more than 50% of parameter sets can reproduce expected error patterns for both AC ablation and EGF overexpression conditions (Fig 7C and 7D). All three strategies are able to reproduce expected error patterns. However, different types of topologies show different distributions in error patterns. Under the mutant condition of AC ablation, the most frequent error pattern is 3°3°3°3°3°. Except 3°3°3°3°3° pattern, topologies with sequential module (1P-5P) predominantly produce 3°2°3°2°3° and 3°2°2°2°3 patterns; topologies with morphogen gradient strategy mainly produce 3°2°3°2°3° and 3°3°1°3°3° patterns; while topologies with the lateral antagonism strategy mainly produce 3°2°3°2°3° pattern (Fig 7C and 7E). Under the mutant condition of EGF overexpression, topologies with sequential module mainly output 1°1°1°1°1° pattern; topologies with morphogen gradient strategy mainly produce 1°1°1°1°1° and 2°1°1°1°2° pattern; and lateral antagonism strategy tends to produce 1°2°1°2°1° pattern (Fig 7D and 7E). Being able to reproduce expected error patterns suggests all three strategies exist in the real biology systems.

We also investigated the performance of identified topologies under the condition of altered lateral signaling. We studied the cell fate patterns performed by topologies that have the link 5P in the mutation of decreased and increased lateral induction capability, which partially mimics the mutation of Notch signaling. We observed expected error patterns for examined topologies as reported in previous study [27]: the loss of 2° fate in 2° cells for decreased lateral induction capability, and the specification of 2° fate in 3° cells for increased lateral induction capability (S6A Fig). Next, we investigated pattern formation by the topologies with lateral antagonism strategy under the mutation of lateral inhibition capability for 10N. For decreased lateral inhibition capability, more than half of the simulations perform the error pattern with 2° fate specified in 1° cell, meaning the failure of the inhibition of 2° fate in 1° cell (S6B Fig). In contrast, increasing the lateral inhibition capability causes the defect of the 2° fate specification in 2° cells.

### Mapping the biological network

**Fig 8:**
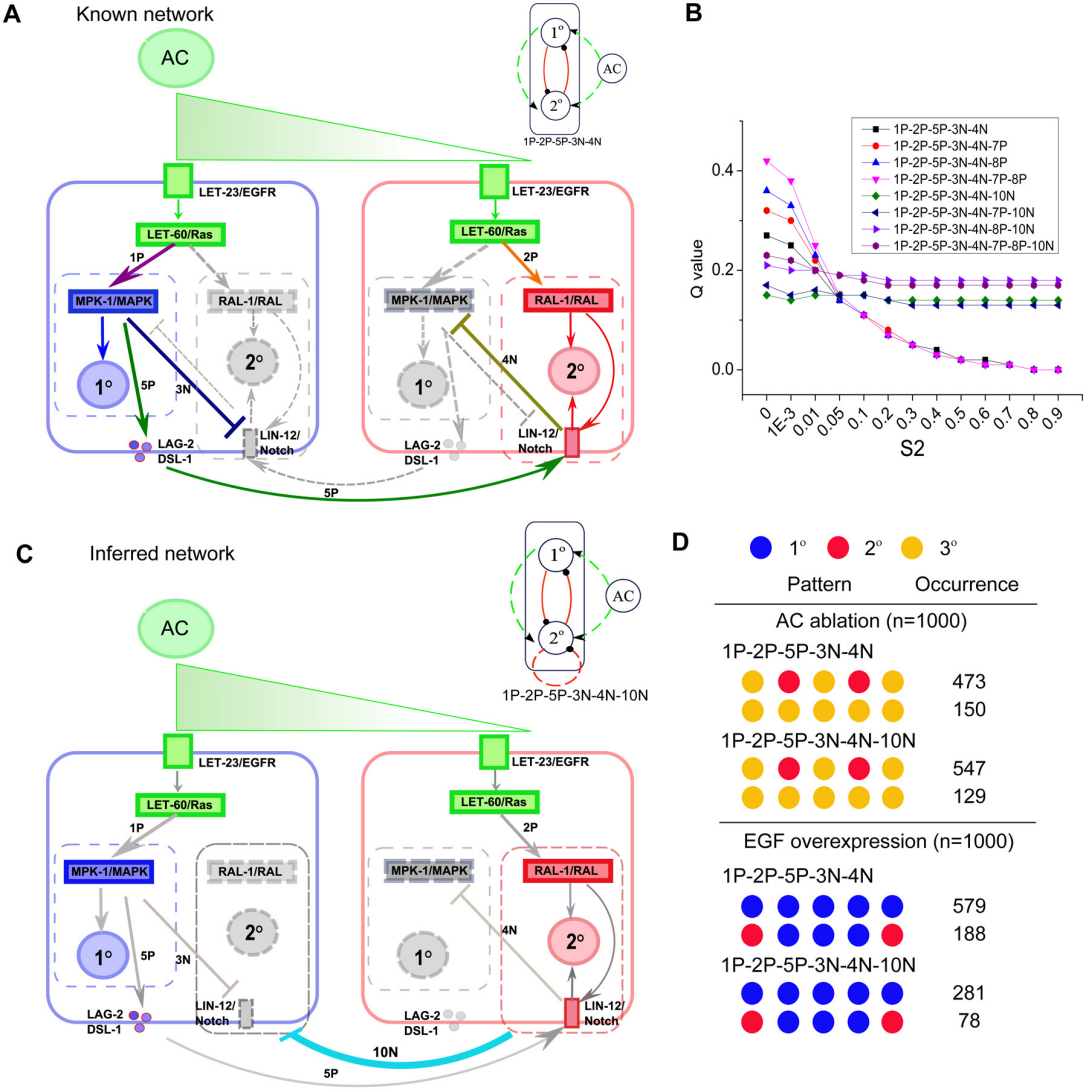
Biological network and its mapping to 1P-2P-5P-3N-4N. (A) The key known pathways in VPC patterning. The proteins and pathways in green correspond to the AC node; those in blue correspond to the 1° node; those in red correspond to the 2° node; and gray indicates that they are repressed in that cell. The regulations among different nodes are labeled with the corresponding links in the topology 1P-2P-5P-3N-4N and in different colors. (B) *Q* values of the topologies that contain 1P-2P-5P-3N-4N. (C) Inferred network constructed based on known links and our inferred link 10N, which is an inhibitory regulation between the 2° nodes of neighboring Pn.p cells. The known links are in gray. (D) Mutant patterns produced by topologies 1P-2P-5P-3N-4N and 1P-2P-5P-3N-4N-10N under AC ablation and EGF overexpression. The top two most-frequent patterns along with their occurrences from total 1,000 runs of simulation are shown. Simulation was modeled with “Combined AND & Additive” rule.

We then asked what topology is the closest to the underlying biological network that executes VPC patterning. We searched the literature and mapped the known pathways that have experimental supports to the simplified links in our coarse-grained model (Fig 8A). In the sequential induction model, EGF signaling from the AC induces the 1° fate in the 1° cell through the Ras-MAPK pathway, and then MAPK promotes Delta-Notch lateral signaling among Pn.p cells to specify 2° fates in 2° cells [14,28]. As a support to the morphogen-based model, the 2° fate in the 2° cells have also been found to be induced by EGF signaling through the RGL-1-RAL-1 pathway [22]. In addition, the negative regulatory roles in the crosstalk between the MAPK and Notch pathways have been found in VPC pattern formation [18,29]. To map these pathways to our 2-node model, MAPK and the downstream pathways are represented by the 1° node, while Ral, Notch and their downstream pathways are represented by the 2° node. Thus, we recovered the topology 1P-2P-5P-3N-4N by mapping the regulatory links among these nodes (Fig 8A). Previous experiments have shown that VPCs lacking *let-23* or other components of the Ras-MAPK pathway can specify the 2° fate, as long as they are adjacent to a 1° VPC [19], which means that the specification of 2° fate can be induced by 5P without 2P. On the other hand, evidences also show that an isolated VPC can adopt a 2° fate if exposed to an intermediate LIN-3 concentration [12], which implies that the induction of 2° fate can be induced by 2P independent of 5P. In addition, Zand et al. reported that RAL-1 is sufficient to promote Notch pathway activity and RAL-1 cooperates with Notch to specify 2° fate [22], which suggests 2P is sufficient to specify 2° cell fate. Since 2P and 5P are both sufficient for 2° fate specification, we examined the *Q* value of this topology with the “Combined AND and Additive” rule. The *Q* values of the topology 1P-2P-5P-3N-4N for S2 ≤ 0.1 are all >0.1.

Notably, this topology agrees with the experimental results for both sequential and morphogen-based induction models [12,19]. This can be tested by investigating special conditions as follows. In the experiments that support the sequential induction model, signaling from AC to the 2° cells is not required for 2° fate in the 2° cells [12]. Accordingly, we checked the *Q* value of the topology 1P-2P-5P-3N-4N in the condition of S2 = 0, and found that the topology is able to robustly (*Q* = 0.27) execute the cell fate patterning, consistent with the experiment result [12]. In the experiments that support morphogen-based model, single VPCs are isolated to avoid intercellular actions (Delta-Notch signaling) among Pn.p cells [12]. Accordingly, we remove the link 5P from the topology to simulate the assay of isolating VPCs. We found that 1P-2P-3N-4N can also perform the cell fate patterning for medium S2 levels (S2 Table).

We then asked why the wild type cells choose this biological network while its *Q* value is not the highest among all the identified topologies in our analysis. One potential advantage of this network is that this network combines both the sequential induction motif 1P-5P and the morphogen gradient motif 1P-2P-3N. This combination makes it possible to execute sequential induction in the case of low S2 and could also use morphogen gradient strategy in the case of certain disruptions such as the disturbance in Delta-Notch signaling. In this regard, the network is more robust than the topologies with either only sequential induction motif or only morphogen gradient motif.

Equipped with our analysis of all functional topologies, we asked whether there might be undiscovered links in the underlying biological network. To address this question, we collected all the functional topologies that contain 1P-2P-5P-3N-4N, and found that adding the self-regulating links 7P and 8P increases the *Q* value under most conditions (Fig 8B). Other than 7P and 8P, 10N is the only additional link among those topologies. Interestingly, with the link 10N, the topology has the potential to use the lateral antagonism strategy, which greatly increases the robustness of the topology for high S2—without 10N, these topologies cannot achieve the patterning function for high S2 (Fig 8B). In contrast, the *Q* values for the topologies with 10N decrease with low S2, but are still >0.1. As a result, the topologies with 1P-2P-5P-3N-4N-10N have stable *Q* values for a broad range of S2, which would make the system much more robust to variations in AC signal. Therefore, we predict that 10N may exist in at least some real systems (Fig 8C). Note that both topologies, 1P-2P-5P-3N-4N (Fig 8A) and 1P-2P-5P-3N-4N-10N (Fig 8C), are able to reproduce the experimental error patterns [26] under the simulated mutation of AC ablation and EGF overexpression (Fig 8D). Interestingly, comparing with the topology 1P-2P-5P-3N-4N, the predicted topology 1P-2P-5P-3N-4N-10N has the potential to use all three patterning strategies. The existence of 10N link can be tested: if experiments under higher S2 conditions show that the network still achieves the VPC patterning, we suggest that there may be an unknown counteracting regulation among the 2° nodes of different Pn.p cells (Fig 8C).

## Discussion

Developmental robustness of the *C*. *elegans* vulval patterning process to a variety of genetic and environmental perturbations has been of long-standing interest. Previous experimental studies have focused on characterizing robustness by measuring the variation of vulval traits under stochastic, environmental or genetic perturbations, and determining the range of variation that allows the formation of a wild-type cell fate pattern [20,27,30]. In our study, we reversely use the robustness property of VPC fate patterning to infer the network and design principle of the system. We used varying parameters to define the system and searched robust topologies that output wild type cell fate pattern in a considerable range of parameter space. Using this method, we successfully recovered the known sequential and morphogen gradient induction strategies and further predicted a novel lateral antagonism strategy that robustly performs the VPC fate patterning. Although different strategies show robustness in different ranges of AC signaling, these ranges overlap. Furthermore, we show that some top topologies (e.g. topologies with module 1P-2P-5P) have the modules to use two or three strategies. These topologies are more robust in various conditions of AC signaling than those that only use one specific strategy. In addition, the constraint of signaling conditions for different patterning strategies and the error patterns emerged by simulated mutation suggest the limitations of the robustness of the system.

Our analysis reveals three basic strategies for VPC fate patterning, providing new insights of the complex system. One of the most debated questions about VPC patterning has been the contribution of EGF versus Notch signaling towards 2° fate formation. Hoyos and colleagues proposed a DSL-Notch autocrine loop to explain the isolated 2° fate specification at intermediate EGF levels [21]. However, they did not find the direct regulation from EGF signaling to 2° fate. More recently, Zand et al. found that EGF signaling can directly contribute to 2° fate through RGL-1-RAL-1 pathway [22,23]. RGL-1-RAL-1 was found to antagonize Ras-Raf pro-1° fate activity, and RAL-1 was found to be sufficient to promote Notch activity and to cooperate with Notch to promote 2° fate. This new Ras effector-switching mechanism provides a new explanation of the morphogen gradient-determined cell fate patterning. There are still unknown questions. For example, how do different source signals determine the different outputs of pro-1° effector or pro-2° effector in originally identical cells? Zand et al. showed that regulation of RAL-1 expression provides a mechanism of switch between pro-1° and pro-2° effectors [22]. But how different source signals determine the different expressions of RAL-1 in presumptive 1° and 2° cells that are exactly the same before induction is still unknown. Due to the coarse-grained nature, our model cannot provide details of the molecular mechanism, but let us try here to give some clues based on our studies. Besides the sequential induction strategy, our analysis found two strategies both with module 1P-2P. The first one, morphogen gradient strategy, mainly relies on the graded AC signal input to the presumptive 1° and 2° cells, and provides a concise principle, i.e. while low source signaling level is sufficient for the specification of 2° fate, the specification of 1° fate requires a high source signaling. Following this principle and the previous observation of the dynamic expression of RAL-1 in Pn.p cells [22], we hypothesize that in the early vulval development, intermediate EGF dose induces the expression of RAL-1 in 1° and 2° cells, and then high EGF dose input to 1° cell turns on the pro-1° pathway activity and inhibits the expression of RAL-1. Thus the expression of RAL-1 is only restricted to 2° cells. Another strategy, lateral antagonism strategy, introduces a new approach to the cell fate patterning, which relies on the lateral antagonism from the 2° fate activity in 2° cells against the induction of 2° fate activity in 1° cell. Following this strategy, we hypothesize that in the early vulval development, pro-2° activity is promoted through RGL-1-RAL-1 pathway by EGF signaling in the presumptive 1° and 2° cells. Then the activated 2° fate regulates the expression of some unknown molecule that is secreted and functions as an antagonist to repress the 2° fate activity in adjacent cells. One possibility is that the antagonist competes against the ligands of Notch pathway and represses the Notch pathway dependent 2° fate specification in the adjacent cells. In this competition, it is highly likely that 2° cells win because there are two 2° cells on the two sides of 1° cell while only one 1° cell on one side of each of 2° cells. The observation that higher ratio of membrane-bound to diffusible intercellular regulation results in higher *Q* values of the topologies with lateral antagonism strategy (Fig 6) suggests the antagonist is likely to function in the membrane-bound way. Another possibility of the inhibitory regulation from activated 2° fate against the 2° fate in adjacent cells is the competition of Notch receptor for the limited ligands between adjacent Pn.p cells.

In this study, we simulated models using different ranges of source signals from the AC, and found that the levels of these signals result in different kinds of topologies and patterning strategies that robustly perform the patterning function. In practice, it is difficult to determine the levels of the source signal in the biological environment. Furthermore, we found that all the three strategies are able to reproduce the expected error patterns in the simulation of mutant AC signaling. Therefore, we cannot rule out the existence of any identified strategy in the real system. It is possible that the biological system employs all three strategies to face the varying environment, just as our predicted topology 1P-2P-5P-3N-4N-10N does. It is also possible that the contributions of different strategies differ among different *Caenorhabditis* species. It would be interesting to investigate the differences of the relative ranges of AC signaling for wild type patterns among different *Caenorhabditis* species.

We also simulated different ratios of the membrane-bound (e.g. LAG-2) and diffusible (e.g. DSL) signals in the intercellular regulation. Previous work has shown that secreted and transmembrane ligand proteins are redundant in *C*. *elegans* vulval development [17]. In this work, we found that the topologies are more robust for a higher proportion of membrane-bound intercellular regulation than for a lower proportion, especially for top topologies with higher S2, which may suggest that the transmembrane intercellular regulation is more important.

Corson and Siggia have studied an elegant mathematical model of the VPC patterning [31]. Based on existing experimental data for VPC patterning in diverse genetic backgrounds as well as timed ablation of the inductive signal, they constructed a “geometric model”. It would be interesting to investigate the relationship between their model and our model, and in particular the manifestation of the distinct patterning strategies in the two models.

## Materials and Methods

### The ODE model

We modeled the process of the 1° and 2° nodes in five cells (Pn4.p to Pn.8) given an input signal from the AC. For a given topology, each cell has the same nodes and links. The regulation of each link is modeled with the Hill function. For example, a link from A has either the form *A* ^*n*^/(*k* ^*n*^+*A* ^*n*^) (positive regulation) or *k* ^*n*^/(*k* ^*n*^+*A* ^*n*^) (negative regulation), where A has two sources: inside or outside the cell. If the source of A is outside the cell, A is calculated as the weighted average of the average concentration of the membrane-bound source in the neighboring cells and the average concentration of the diffusible source from neighboring cells and the cell itself. Each node can be regulated by multiple links. For the “AND” rule, all the regulatory links to the same node are modeled as their product. For the “Combined AND and Additive” rule, positive regulatory links to the same node are modeled as their weighted sum, and negative regulatory links are modeled as their product. Each node has a half-life *τ*. After normalization, as in previous work [5], each node has one parameter *τ* and each link has two parameters (*n* and *k*).

### Nondimensionalization procedure

We used the methods of a previous study [5] to reduce the number of parameters in the Hill functions as follows. If node *A* positively regulates *B* and *B* positively regulates *A*, the rate equations are:

$$\begin{array}{ll} \frac{dA}{dt} & =V_{A}\frac{B^{n_{BA}}}{B^{n_{BA}}+k_{BA}{}^{n_{BA}}}-\frac{A}{\tau_{A}}\\ \frac{dB}{dt} & =V_{B}\frac{A^{n_{AB}}}{A^{n_{AB}}+k_{AB}{}^{n_{AB}}}-\frac{B}{\tau_{B}}. \end{array}$$

We first transform the equations to:

$$\begin{array}{ll} \frac{dA}{dt}\frac{1}{V_{A}\tau_{A}} & =\frac{1}{\tau_{A}}(\frac{B^{n_{BA}}}{B^{n_{BA}}+k_{BA}{}^{n_{BA}}}-\frac{A}{V_{A}\tau_{A}})\\ \frac{dB}{dt}\frac{1}{V_{B}\tau_{B}} & =\frac{1}{\tau_{B}}(\frac{A^{n_{AB}}}{A^{n_{AB}}+k_{AB}{}^{n_{AB}}}-\frac{B}{V_{B}\tau_{B}}). \end{array}$$

Let $\frac{A}{V_{A}\tau_{A}}\Rightarrow A,\frac{B}{V_{B}\tau_{B}}\Rightarrow B,\frac{k_{BA}}{V_{B}\tau_{B}}\Rightarrow k_{BA},\frac{k_{AB}}{V_{A}\tau_{A}}\Rightarrow k_{AB}$, we get:

$$\begin{array}{ll} \frac{dA}{dt} & =\frac{1}{\tau_{A}}(\frac{B^{n_{BA}}}{B^{n_{BA}}+k_{BA}{}^{n_{BA}}}-A)\\ \frac{dB}{dt} & =\frac{1}{\tau_{B}}(\frac{A^{n_{AB}}}{A^{n_{AB}}+k_{AB}{}^{n_{AB}}}-B). \end{array}$$

Thus, each node has a parameter *τ*, and each link has two parameters *n* and *k*.

### Models for the “AND” and “Combined AND and Additive” rules

Denote:

$$H(A,n,k)=\frac{A^{n}}{A^{n}+k^{n}}$$

and

$$G(A,k,n,\delta )=\{\begin{array}{ll} H(A,n,k), & \delta =1\\ 1-H(A,n,k), & \delta =-1 \end{array}$$

where *δ* = 1 represents positive and -1 represents negative regulation from *A*. If node *B* has more than one regulation from node(s) *A*_*i*_, we have different models for the “AND” and “AND and Additive” rules. For the “AND” rule, we used the product of each regulation to model the multiple regulation as:

$$\frac{dB}{dt}=\frac{1}{\tau_{B}}(\underset{i}{\prod }G(A_{i},k_{i},n_{i},\delta_{i})-B).$$

For the “Combined AND and Additive” rule, we have:

$$\frac{dB}{dt}=\frac{1}{\tau_{B}}(\underset{\delta_{i}=-1}{\prod }G(A_{i},k_{i},n_{i},\delta_{i})\cdot \underset{\delta_{i}=1}{\sum }\beta_{\text{i}}G(A_{i},k_{i},n_{i},\delta_{i})-B).$$

### Proportions of diffusible and membrane-bound intercellular regulation among VPCs

If node *B* in one cell *m* is regulated from node *A* intracellularly, the value *A* in the above function *G*(*A*,*k*,*n*,*δ*) is represented as the concentration of *A* in the cell. If regulation from node *A* is intercellular, then the value *A* in the function *G*(*A*,*k*,*n*,*δ*), denoted as *A*_out_, depends on the proportions of diffusible and membrane-bound intercellular regulation among VPCs. The proportion of diffusible intercellular regulation was denoted *r* and membrane-bound regulation as 1-*r* (0 ≤ r ≤ 1), and the concentration of *A* in the adjacent cell(s) *n* was denoted *A*_*n*_, and in the cell *m* as *A*_*m*_. Let *E*_*d*_ be the mean value of all the *A*_*n*_ and *A*_*m*_, and *E*_*b*_ the mean value of all *A*_*n*_, then *A*_out_ is calculated as:

$$A_{\text{out}}=rE_{d}+(1-r)E_{b}.$$

### Simulation

For each of the two logic rules, we generated 10,000 parameter sets using the LHS method [32]. The ranges of the following parameters were selected based on previous studies [5]: *k* = (0.001–1), *n* = (2–10) and *τ* = (5–100 min). *k* was uniformly sampled in the log scale, and both *n* and *τ* were in the linear scale. For the “Combined AND and Additive” rule, the parameters of the weight for each positive regulation were evenly sampled with the range of [0, 1]. We used the GNU Scientific Library for ODE simulation as in previous publications [5,33]. Calculation time was set at 2000 min (virtual simulation time), which is long enough for the patterning system to reach steady state. For each logic rule and for each of the S2 values (S2 = 0, 0.001, 0.01, 0.05, 0.1, 0.2, …, 0.9), we enumerated all the possible topologies for the ODE simulation. For the general simulation, the proportion of membrane-bound and diffusible intercellular actions was randomly selected from the range [0, 1].

### Modeling the VPC patterning

We constructed a model of the anchor cell (AC) and five Pn.p cells (P4.p –P8.p), each having two nodes (1° and 2°). Given the input signal from the AC to the closest Pn.p cell (P6.p), denoted S1, and to VPCs at intermediate distances (P5.p and P7.p), denoted S2, and initial values of both nodes in all cells as 0, we modeled the variation of the values of the two nodes in all VPCs within a given time period. Each node in the VPCs could be regulated from the other node and itself intracellularly and intercellularly, and from the AC.

### Determination of patterning function

We used the method as in Ma et al. [5] to judge the patterning function of a topology with a given parameter set. Specifically, let *x*(*I*; *n*) be the value of the node *I* in the cell *n*. The node *I* can be 1° or 2°, and there are five cells in our simulated system. We set the initial state of both 1° and 2° nodes in all the cells as 0, and the target steady-state that the system should reach in the given time as follows: *x*(1; 3) = 1, *x*(2; 2) = 1, *x*(2; 4) = 1, and other values of *x* are 0 (Fig 1B). For node *I* in cell *n*, a score *T* is given to evaluate whether its expression level is consistent with the target pattern:

$$\begin{array}{ll} T_{off} & =\alpha_{\text{max}}f(x,(I,n))=\alpha_{\text{max}}\frac{{\left(x,(I,n)/x_{t}\right)}^{3}}{1+{\left(x,(I,n)/x_{t}\right)}^{3}},\\ T_{on} & =\alpha_{\text{max}}(1-f(x,(I,n))), \end{array}$$

where *x*_*t*_ is the threshold for *x* (10% here) and *α*_max_ the worst-possible score (0.5 here). *T*_*off*_ is used when the target state has a low value (0), and *T*_*on*_ is used for a target state with a high value (1). The total score is calculated as:

$$\underset{node}{\sum }\underset{cell}{\sum }T(x,(I,n)).$$

If the total score is <0.0125, the pattern is accepted.

### Simulation of mutant conditions

To simulate the AC ablation and EGF overexpression, for each topology, we first chose the best AC signal condition under which that topology has the highest *Q* value. Then we randomly selected 1,000 functional parameter sets that output wild type pattern. To simulate the AC ablation, we set AC signal to 0 during the simulation, we tried different time points (1 min, 10 min, 100 min, 200 min) to eliminate the signal, and selected 100 min as the best condition according to similarity between output patterns and expected patterns. To simulate the EGF overexpression, we tried several conditions of high AC signal level, and we selected the condition of S1 = 1, S2 = 0.5, S3 = 0.1, which gave the best output patterns. We run the simulation of topology with selected parameters under the conditions of AC ablation and EGF overexpression and calculated the frequency to generate expected patterns.

To simulate the mutation of lateral signaling, we also chose the best signal condition and 1,000 functional parameter sets as described above, and then, changed the parameter *k* of corresponding link in our models. We increased the parameter *k* of 5P, which mimics lateral Notch signaling, to 10 times of the original value to simulate the decrease of the lateral induction capability of 5P, and decreased *k* to one tenth of the original value to simulate the increase of the capability. Similarly, we increased the parameter *k* of 10N to 10 times to simulate the decrease of lateral inhibition capability of 10N, and decreased *k* to one tenth to simulate the increase of the capability.

We calculated the average frequencies of error patterns output by topologies with different patterning strategies under the mutant conditions. For sequential induction strategy, all the topologies that have the module 1P-5P are counted. For lateral antagonism strategy, all the topologies that have module 1P-2P and the link 10N are counted. For morphogen gradient strategy, all the topologies that have module 1P-2P but without 10N are counted.

## Supporting Information

S1 Fig The spaces of functional topologies with “AND” rule. The spaces of functional topologies with “AND” rule for S2 = 0 (A), 0.1 (B), and 0.5 (C) are shown. Each node represents a functional topology, with its size corresponding to the *Q* value of the topology. The links between two nodes mean that the two topologies share parameter sets, and the number of shared parameter sets is reflected by the line thickness. The top topologies are labeled with their names and in different colors (green for topologies with motif 1P-5P and blue for topologies with motif 1P-2P).
(TIF)

S2 Fig The strategy used by 1P-2P-3N-6P with “AND” rule. This strategy works only with “AND” rule. A simple description of the strategy, representative topology, and the S2 level are listed in the table. Below the table shows the mechanism of representative topology: on the left is a sketch of the topology; in the middle is the graph that shows the regulation among the AC, 1°, and 2° nodes in the 1° (middle) and 2° cells (two sides), where the heavy full lines indicate acting or strong regulation and fine dashed lines indicate no or weak regulation; on the right draws the dynamical value of each node in the 1° cell and 2° cells with increasing time.
(TIF)

S3 Fig Clustering of top topologies with *Q* values for different S2. (A) “AND” rule; (B) “Combined AND & Additive” rule. Left: topological clustering results; right: *Q* values for each corresponding topology from the clustering graph for different S2. The topologies with *Q* values ranking top 5 for at least one S2 are selected.
(TIF)

S4 Fig High structural flexibility in robust topologies. Plots of topologies with their structural flexibility and functional robustness reveal high structural flexibility in robust topologies. Plots for different S2 (from left to right: 0.001, 0.01, 0.1, and 0.5) and with both the “AND” and “Combined AND & Additive” rules are shown.
(TIF)

S5 Fig Simulation under the conditions of varying S1 and S2 concordantly. (A) Plots of *Q* values of three topologies 1P-5P-2N-4N (sequential induction), 1P-2P-3N (morphogen gradient), 1P-2P-4N-10N (lateral antagonism) under the increased AC signal (2× and 5×). The basal condition is S1 = 1, S2 = 0.1, S3 = 0. (B) Plot of *Q* values of three topologies under the condition of decreased AC signal (0.5× and 0.1×). The basal condition is S1 = 1, S2 = 0.1, S3 = 0. Both S1 and S2 increase concordantly.
(TIF)

S6 Fig Simulation of top topologies under the mutant conditions of lateral signaling. (A) Patterns produced by topologies with sequential module (1P-5P) under the mutant conditions of decreased (left) and increased (right) lateral induction capability for the 5P link. (B) Patterns produced by topologies with “lateral antagonism” strategy (1P-2P-4N-10N) under the mutant conditions of decreased (left) and increased (right) lateral inhibition capability for the 10N link. The top two most-frequent patterns along with corresponding ratios of 1,000 runs of simulation are shown. Simulation was modeled with “Combined AND & Additive” rule.
(TIF)

S1 Table *Q* values of topologies for different S2 with the “AND” rule. (DOCX)

S2 Table *Q* values of topologies for different S2 with the “Combined AND & Additive” rule. (DOCX)

S3 Table *Q* values of top topologies for different S2 with the “AND” rule. (DOCX)

S4 Table *Q* values of top topologies for different S2 with the “Combined AND & Additive” rule. (DOCX)

S5 Table *Q* values of topologies for different ratios of diffusible to membrane-bound intercellular regulation for S2 = 0. (DOCX)

S6 Table *Q* values of topologies for different ratios of diffusible to membrane-bound intercellular regulation for S2 = 0.01. (DOCX)

S7 Table *Q* values of topologies for different ratios of diffusible to membrane-bound intercellular regulation for S2 = 0.1. (DOCX)

S8 Table *Q* values of topologies for different ratios of diffusible to membrane-bound intercellular regulation for S2 = 0.5. (DOCX)

S9 Table *Q* values of top topologies with different AC signal levels with “AND” rule. (DOCX)

S10 Table *Q* values of top topologies with different AC signal levels with “Combined AND & Additive” rule. (DOCX)

S11 Table Topologies with frequencies of expected error patterns under simulated mutant AC signaling with “AND” rule. (DOCX)

S12 Table Topologies with frequencies of expected error patterns under simulated mutant AC signaling with “Combined AND & Additive” rule. (DOCX)

S1 Text The constraint of parameter sets for 1P-2P-3N to function using a morphogen gradient strategy. (DOCX)
