# Supplementary figures and images for: An Atlas of Network Topologies Reveals Design Principles for Caenorhabditis elegans Vulval Precursor Cell Fate Patterning
Source: PLoS One. 2015 Jun 26;10(6):e0131397. doi: 10.1371/journal.pone.0131397 (PMC4482679; doi:10.1371/journal.pone.0131397)

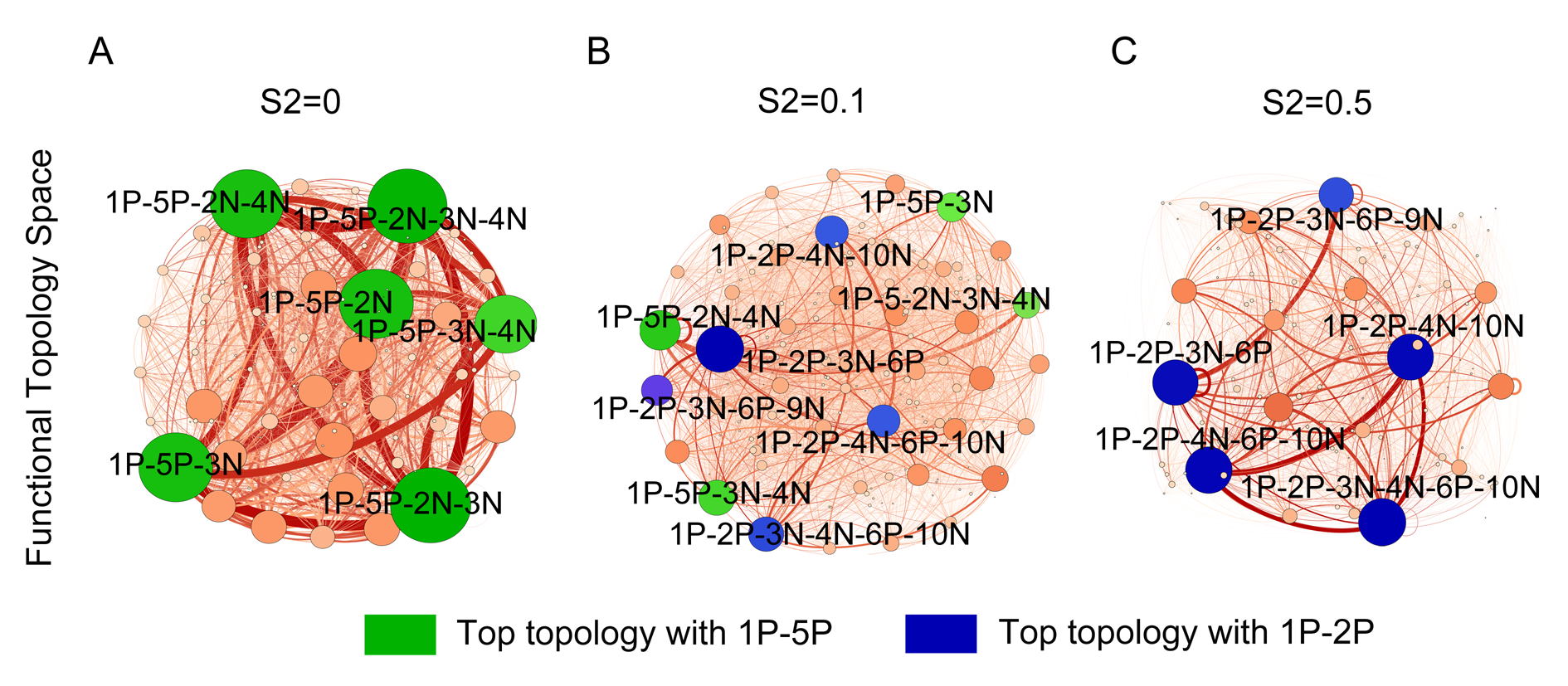

Supplement: S1 Fig — The spaces of functional topologies with “AND” rule for S2 = 0 (A), 0.1 (B), and 0.5 (C) are shown. Each node represents a functional topology, with its size corresponding to the Q value of the topology. The links between two nodes mean that the two topologies share parameter sets, and the number of shared parameter sets is reflected by the line thickness. The top topologies are labeled with their names and in different colors (green for topologies with motif 1P-5P and blue for topologies with motif 1P-2P). (TIF) [file pone.0131397.s001.tif]

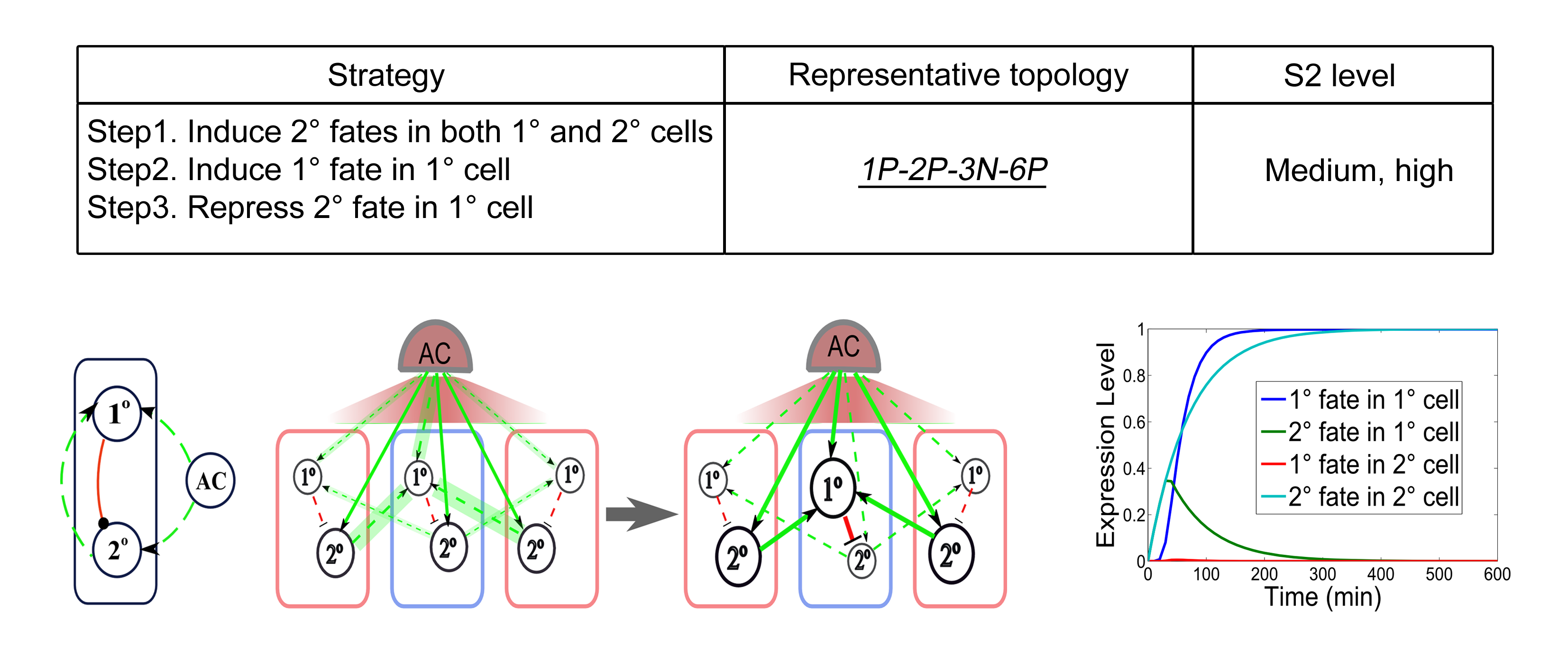

Supplement: S2 Fig — This strategy works only with “AND” rule. A simple description of the strategy, representative topology, and the S2 level are listed in the table. Below the table shows the mechanism of representative topology: on the left is a sketch of the topology; in the middle is the graph that shows the regulation among the AC, 1°, and 2° nodes in the 1° (middle) and 2° cells (two sides), where the heavy full lines indicate acting or strong regulation and fine dashed lines indicate no or weak regulation; on the right draws the dynamical value of each node in the 1° cell and 2° cells with increasing time. (TIF) [file pone.0131397.s002.tif]

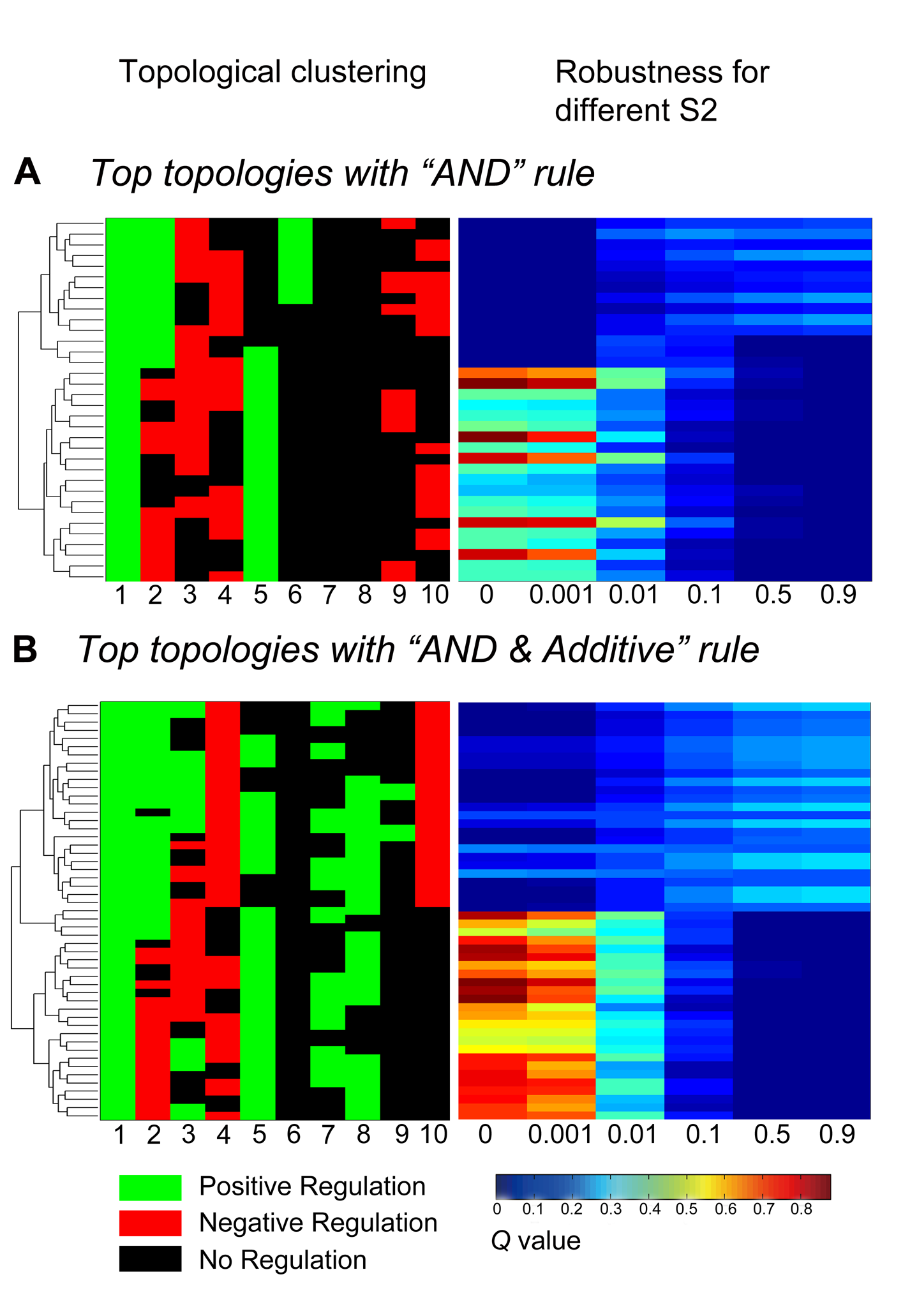

Supplement: S3 Fig — (A) “AND” rule; (B) “Combined AND & Additive” rule. Left: topological clustering results; right: Q values for each corresponding topology from the clustering graph for different S2. The topologies with Q values ranking top 5 for at least one S2 are selected. (TIF) [file pone.0131397.s003.tif]

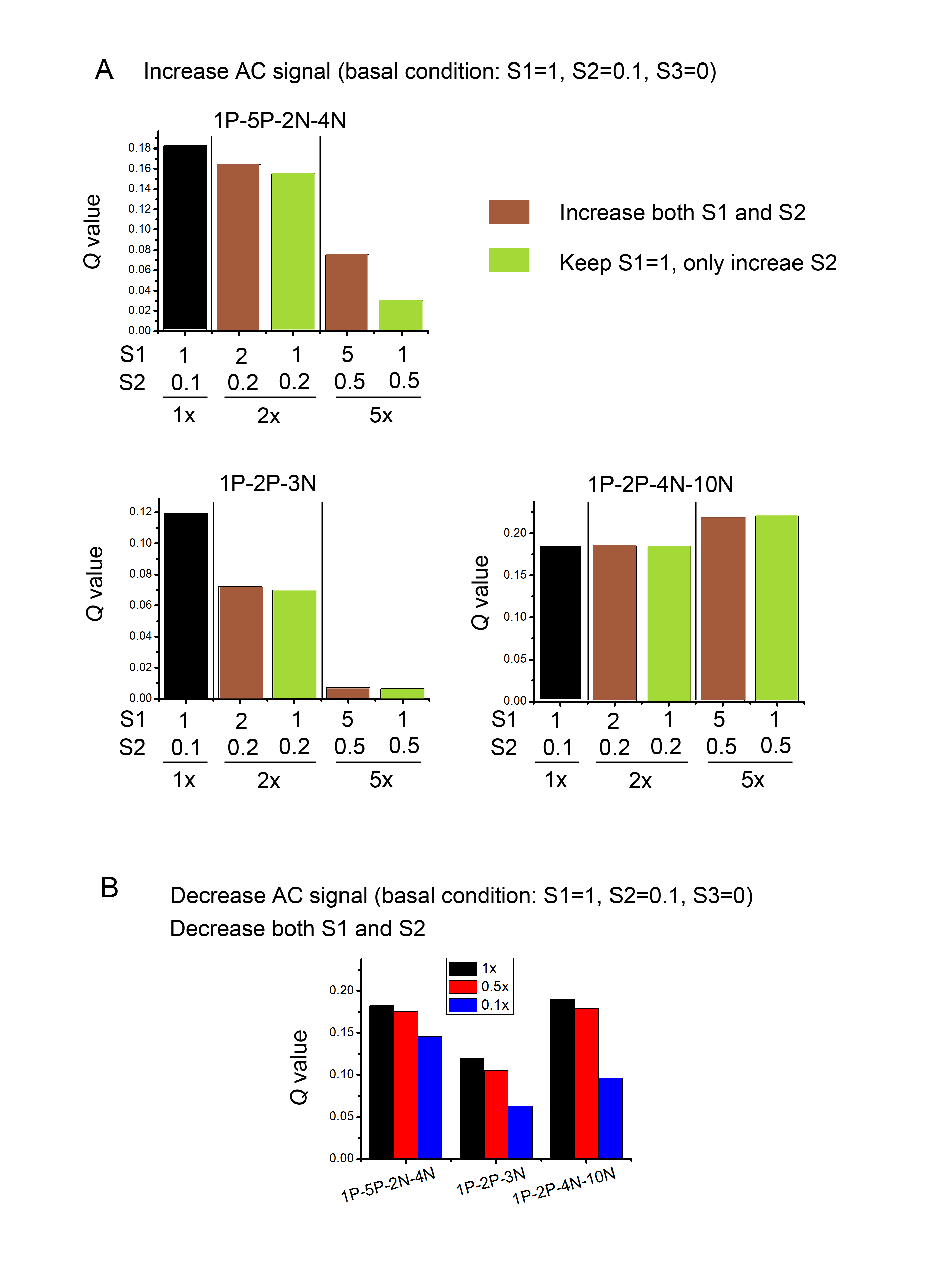

Supplement: S5 Fig — (A) Plots of Q values of three topologies 1P-5P-2N-4N (sequential induction), 1P-2P-3N (morphogen gradient), 1P-2P-4N-10N (lateral antagonism) under the increased AC signal (2× and 5×). The basal condition is S1 = 1, S2 = 0.1, S3 = 0. (B) Plot of Q values of three topologies under the condition of decreased AC signal (0.5× and 0.1×). The basal condition is S1 = 1, S2 = 0.1, S3 = 0. Both S1 and S2 increase concordantly. (TIF) [file pone.0131397.s005.tif]

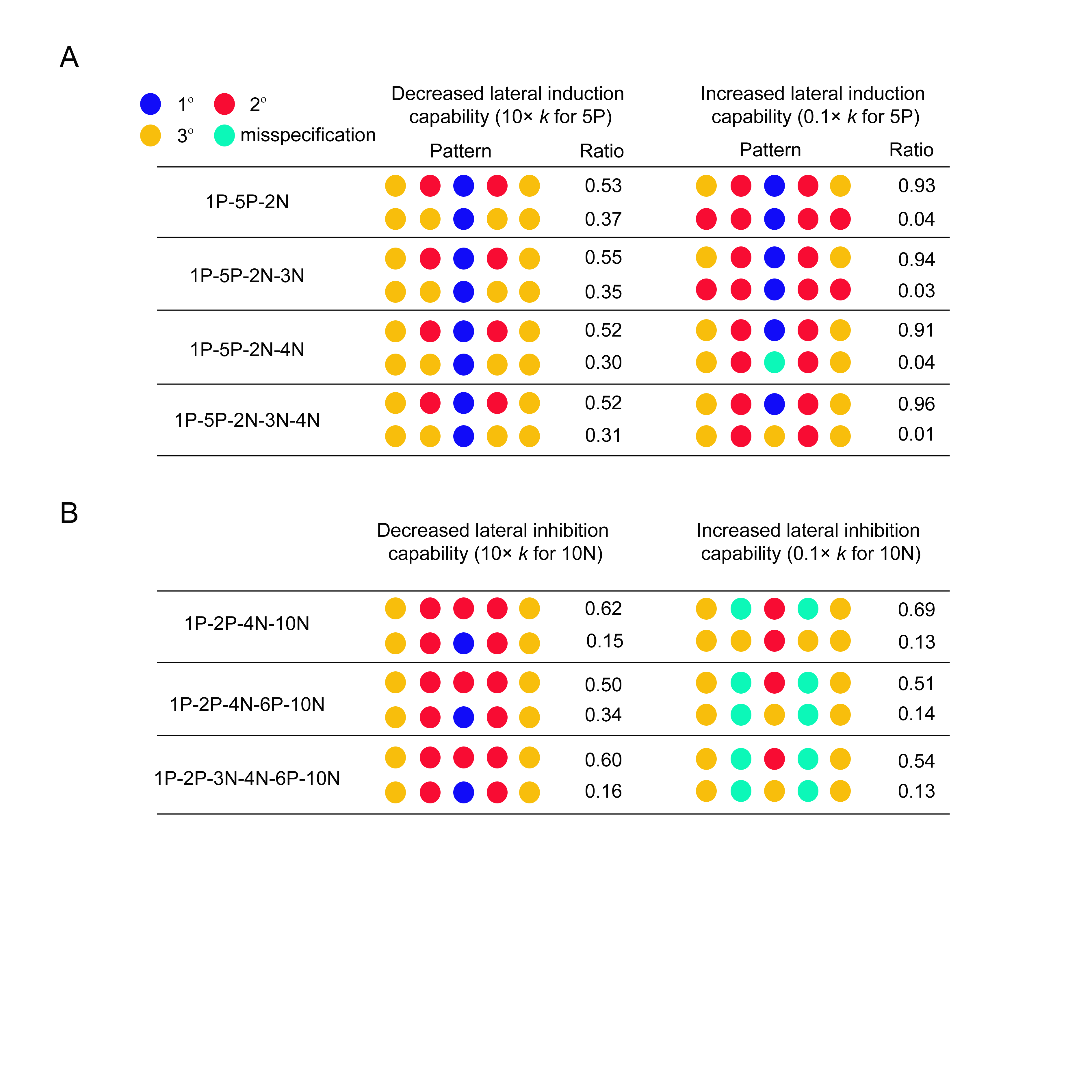

Supplement: S6 Fig — (A) Patterns produced by topologies with sequential module (1P-5P) under the mutant conditions of decreased (left) and increased (right) lateral induction capability for the 5P link. (B) Patterns produced by topologies with “lateral antagonism” strategy (1P-2P-4N-10N) under the mutant conditions of decreased (left) and increased (right) lateral inhibition capability for the 10N link. The top two most-frequent patterns along with corresponding ratios of 1,000 runs of simulation are shown. Simulation was modeled with “Combined AND & Additive” rule. (TIF) [file pone.0131397.s006.tif]
