# Supplementary material for: An Atlas of Network Topologies Reveals Design Principles for Caenorhabditis elegans Vulval Precursor Cell Fate Patterning
Source: PLoS One. 2015 Jun 26;10(6):e0131397. doi: 10.1371/journal.pone.0131397 (PMC4482679; doi:10.1371/journal.pone.0131397)
Supplement: S1 Table — (DOCX) [file pone.0131397.s007.docx]

**S1 Table. *Q* values of topologies for different S2 with the “AND” rule.**

| Topology | S2 | | | | | | | | | | | | |
| --- | --- | --- | --- | --- | --- | --- | --- | --- | --- | --- | --- | --- | --- |
|  | 0 | 0.001 | 0.01 | 0.05 | 0.1 | 0.2 | 0.3 | 0.4 | 0.5 | 0.6 | 0.7 | 0.8 | 0.9 |
| 1P-5P-2N-4N | 0.78 | 0.77 | 0.46 | 0.26 | 0.18 | 0.10 | 0.06 | 0.03 | 0.02 | 0.01 | 0.00 | 0.00 | 0.00 |
| 1P-5P-2N-3N-4N | 0.84 | 0.80 | 0.40 | 0.20 | 0.12 | 0.07 | 0.04 | 0.03 | 0.02 | 0.01 | 0.01 | 0.00 | 0.00 |
| 1P-5P-3N | 0.78 | 0.66 | 0.40 | 0.22 | 0.14 | 0.07 | 0.04 | 0.02 | 0.00 | 0.00 | 0.00 | 0.00 | 0.00 |
| 1P-5P-3N-4N | 0.66 | 0.61 | 0.39 | 0.24 | 0.18 | 0.11 | 0.07 | 0.04 | 0.03 | 0.02 | 0.01 | 0.00 | 0.00 |
| 1P-5P-2N-3N | 0.85 | 0.72 | 0.29 | 0.11 | 0.05 | 0.02 | 0.01 | 0.00 | 0.00 | 0.00 | 0.00 | 0.00 | 0.00 |
| 1P-5P-2N | 0.78 | 0.68 | 0.27 | 0.09 | 0.04 | 0.01 | 0.00 | 0.00 | 0.00 | 0.00 | 0.00 | 0.00 | 0.00 |
| 1P-2P-3N-6P | 0.00 | 0.00 | 0.18 | 0.23 | 0.22 | 0.21 | 0.20 | 0.19 | 0.19 | 0.19 | 0.19 | 0.19 | 0.20 |
| 1P-5P-2N-4N-10N | 0.37 | 0.37 | 0.23 | 0.14 | 0.10 | 0.07 | 0.05 | 0.03 | 0.02 | 0.01 | 0.01 | 0.00 | 0.00 |
| 1P-2P-3N-4N-6P-10N | 0.00 | 0.00 | 0.09 | 0.15 | 0.17 | 0.19 | 0.20 | 0.21 | 0.21 | 0.22 | 0.22 | 0.23 | 0.23 |
| 1P-2P-4N-6P-10N | 0.00 | 0.00 | 0.08 | 0.14 | 0.16 | 0.18 | 0.19 | 0.20 | 0.21 | 0.22 | 0.22 | 0.23 | 0.23 |
| 1P-2P-4N-10N | 0.00 | 0.00 | 0.08 | 0.14 | 0.16 | 0.18 | 0.19 | 0.20 | 0.21 | 0.21 | 0.22 | 0.22 | 0.23 |
| 1P-5P-3N-4N-10N | 0.35 | 0.33 | 0.22 | 0.13 | 0.10 | 0.06 | 0.04 | 0.03 | 0.02 | 0.01 | 0.01 | 0.00 | 0.00 |
| 1P-5P-3N-9N | 0.36 | 0.33 | 0.21 | 0.13 | 0.10 | 0.06 | 0.04 | 0.02 | 0.01 | 0.01 | 0.00 | 0.00 | 0.00 |
| 1P-5P-2N-4N-9N | 0.37 | 0.36 | 0.21 | 0.12 | 0.08 | 0.04 | 0.02 | 0.01 | 0.01 | 0.00 | 0.00 | 0.00 | 0.00 |
| 1P-5P-2N-3N-4N-9N | 0.40 | 0.39 | 0.19 | 0.09 | 0.06 | 0.03 | 0.02 | 0.01 | 0.01 | 0.00 | 0.00 | 0.00 | 0.00 |
| 1P-5P-2N-3N-4N-10N | 0.38 | 0.36 | 0.18 | 0.09 | 0.06 | 0.04 | 0.02 | 0.02 | 0.01 | 0.01 | 0.00 | 0.00 | 0.00 |
| 1P-5P-3N-10N | 0.38 | 0.32 | 0.19 | 0.11 | 0.07 | 0.04 | 0.02 | 0.01 | 0.00 | 0.00 | 0.00 | 0.00 | 0.00 |
| 1P-5P-3N-4N-9N | 0.31 | 0.30 | 0.19 | 0.12 | 0.09 | 0.06 | 0.04 | 0.03 | 0.02 | 0.01 | 0.01 | 0.00 | 0.00 |
| 1P-5P-2N-3N-9N | 0.40 | 0.37 | 0.16 | 0.07 | 0.04 | 0.01 | 0.01 | 0.00 | 0.00 | 0.00 | 0.00 | 0.00 | 0.00 |
| 1P-5P-4N-10N | 0.26 | 0.26 | 0.18 | 0.12 | 0.09 | 0.07 | 0.05 | 0.04 | 0.04 | 0.03 | 0.02 | 0.02 | 0.01 |
| 1P-2P-3N-6P-9N | 0.00 | 0.00 | 0.10 | 0.14 | 0.15 | 0.15 | 0.15 | 0.15 | 0.15 | 0.15 | 0.15 | 0.15 | 0.15 |
| 1P-5P-2N-9N | 0.37 | 0.35 | 0.14 | 0.06 | 0.03 | 0.01 | 0.00 | 0.00 | 0.00 | 0.00 | 0.00 | 0.00 | 0.00 |
| 1P-5P-2N-10N | 0.38 | 0.33 | 0.13 | 0.05 | 0.03 | 0.00 | 0.00 | 0.00 | 0.00 | 0.00 | 0.00 | 0.00 | 0.00 |
| 1P-5P-2N-3N-10N | 0.38 | 0.32 | 0.13 | 0.05 | 0.02 | 0.00 | 0.00 | 0.00 | 0.00 | 0.00 | 0.00 | 0.00 | 0.00 |
| 1P-2P-3N-4N-10N | 0.00 | 0.00 | 0.09 | 0.12 | 0.13 | 0.13 | 0.13 | 0.13 | 0.13 | 0.13 | 0.13 | 0.13 | 0.13 |
| 1P-5P-10N | 0.28 | 0.25 | 0.15 | 0.09 | 0.06 | 0.03 | 0.02 | 0.01 | 0.00 | 0.00 | 0.00 | 0.00 | 0.00 |
| 1P-2P-3N-6P-10N | 0.00 | 0.00 | 0.09 | 0.11 | 0.11 | 0.11 | 0.10 | 0.10 | 0.10 | 0.10 | 0.10 | 0.10 | 0.10 |
| 1P-2P-3N-4N-6P | 0.00 | 0.00 | 0.07 | 0.10 | 0.11 | 0.11 | 0.11 | 0.10 | 0.10 | 0.10 | 0.10 | 0.10 | 0.10 |
| 1P-2P-3N-4N-6P-9N-10N | 0.00 | 0.00 | 0.04 | 0.07 | 0.09 | 0.10 | 0.10 | 0.11 | 0.11 | 0.11 | 0.12 | 0.12 | 0.12 |
| 1P-2P-4N-9N-10N | 0.00 | 0.00 | 0.04 | 0.07 | 0.08 | 0.09 | 0.10 | 0.10 | 0.10 | 0.11 | 0.11 | 0.11 | 0.11 |
| 1P-2P-4N-6P-9N-10N | 0.00 | 0.00 | 0.04 | 0.07 | 0.08 | 0.09 | 0.10 | 0.10 | 0.10 | 0.11 | 0.11 | 0.11 | 0.11 |
| 1P-5P-3N-4N-9N-10N | 0.16 | 0.16 | 0.11 | 0.07 | 0.05 | 0.04 | 0.02 | 0.02 | 0.01 | 0.00 | 0.00 | 0.00 | 0.00 |
| 1P-5P-3N-9N-10N | 0.17 | 0.16 | 0.11 | 0.06 | 0.05 | 0.03 | 0.02 | 0.01 | 0.01 | 0.00 | 0.00 | 0.00 | 0.00 |
| 1P-5P-2N-4N-9N-10N | 0.17 | 0.17 | 0.10 | 0.06 | 0.04 | 0.03 | 0.02 | 0.01 | 0.01 | 0.01 | 0.00 | 0.00 | 0.00 |
| 1P-5P-4N | 0.14 | 0.14 | 0.09 | 0.06 | 0.05 | 0.03 | 0.02 | 0.02 | 0.01 | 0.01 | 0.00 | 0.00 | 0.00 |
| 1P-5P-2N-3N-4N-9N-10N | 0.18 | 0.17 | 0.09 | 0.04 | 0.03 | 0.02 | 0.01 | 0.01 | 0.00 | 0.00 | 0.00 | 0.00 | 0.00 |
| 1P-5P-4N-9N-10N | 0.12 | 0.12 | 0.08 | 0.06 | 0.05 | 0.03 | 0.02 | 0.02 | 0.02 | 0.01 | 0.01 | 0.01 | 0.00 |
| 1P-2P-5P-3N-4N | 0.00 | 0.00 | 0.13 | 0.14 | 0.12 | 0.08 | 0.06 | 0.04 | 0.03 | 0.02 | 0.01 | 0.00 | 0.00 |
| 1P-2P-3N | 0.00 | 0.00 | 0.15 | 0.14 | 0.12 | 0.07 | 0.04 | 0.02 | 0.01 | 0.00 | 0.00 | 0.00 | 0.00 |
| 1P-5P-9N-10N | 0.13 | 0.13 | 0.08 | 0.05 | 0.04 | 0.02 | 0.02 | 0.01 | 0.01 | 0.00 | 0.00 | 0.00 | 0.00 |
| 1P-5P-2N-3N-9N-10N | 0.18 | 0.16 | 0.07 | 0.03 | 0.00 | 0.00 | 0.00 | 0.00 | 0.00 | 0.00 | 0.00 | 0.00 | 0.00 |
| 1P-5P-2N-9N-10N | 0.18 | 0.16 | 0.07 | 0.03 | 0.00 | 0.00 | 0.00 | 0.00 | 0.00 | 0.00 | 0.00 | 0.00 | 0.00 |
| 1P-2P-5P-3N | 0.00 | 0.00 | 0.14 | 0.13 | 0.11 | 0.06 | 0.04 | 0.02 | 0.01 | 0.00 | 0.00 | 0.00 | 0.00 |
| 1P-5P | 0.12 | 0.10 | 0.06 | 0.04 | 0.03 | 0.01 | 0.01 | 0.01 | 0.00 | 0.00 | 0.00 | 0.00 | 0.00 |
| 1P-5P-3N-7N | 0.11 | 0.09 | 0.05 | 0.03 | 0.02 | 0.00 | 0.00 | 0.00 | 0.00 | 0.00 | 0.00 | 0.00 | 0.00 |
| 1P-5P-2N-3N-4N-7N | 0.12 | 0.12 | 0.06 | 0.00 | 0.00 | 0.00 | 0.00 | 0.00 | 0.00 | 0.00 | 0.00 | 0.00 | 0.00 |
| 1P-5P-2N-4N-7N | 0.11 | 0.11 | 0.06 | 0.00 | 0.00 | 0.00 | 0.00 | 0.00 | 0.00 | 0.00 | 0.00 | 0.00 | 0.00 |
| 1P-5P-2N-3N-7N | 0.12 | 0.11 | 0.04 | 0.00 | 0.00 | 0.00 | 0.00 | 0.00 | 0.00 | 0.00 | 0.00 | 0.00 | 0.00 |
| 1P-5P-2N-7N | 0.11 | 0.10 | 0.04 | 0.00 | 0.00 | 0.00 | 0.00 | 0.00 | 0.00 | 0.00 | 0.00 | 0.00 | 0.00 |
| 1P-5P-2N-3N-4N-8N | 0.10 | 0.10 | 0.05 | 0.00 | 0.00 | 0.00 | 0.00 | 0.00 | 0.00 | 0.00 | 0.00 | 0.00 | 0.00 |
| 1P-5P-2N-3N-8N | 0.10 | 0.09 | 0.04 | 0.00 | 0.00 | 0.00 | 0.00 | 0.00 | 0.00 | 0.00 | 0.00 | 0.00 | 0.00 |

Only topologies with *Q* ≥ 0.1 for at least one S2 are shown.
