# Supplementary material for: An Atlas of Network Topologies Reveals Design Principles for Caenorhabditis elegans Vulval Precursor Cell Fate Patterning
Source: PLoS One. 2015 Jun 26;10(6):e0131397. doi: 10.1371/journal.pone.0131397 (PMC4482679; doi:10.1371/journal.pone.0131397)
Supplement: S5 Table — (DOCX) [file pone.0131397.s011.docx]

**S5 Table. *Q* values of topologies for different ratios of diffusible to membrane-bound intercellular regulation for S2 = 0.**

|  | Only D | D = 2M | D = M | D = 0.5M | D = 0.01M | Only M |
| --- | --- | --- | --- | --- | --- | --- |
| 1P-5P-2N-3N | 0.81 | 0.84 | 0.85 | 0.85 | 0.87 | 0.87 |
| 1P-5P-2N-3N-4N | 0.80 | 0.82 | 0.83 | 0.84 | 0.87 | 0.87 |
| 1P-5P-2N | 0.73 | 0.75 | 0.77 | 0.78 | 0.85 | 0.87 |
| 1P-5P-2N-4N | 0.71 | 0.74 | 0.76 | 0.78 | 0.86 | 0.87 |
| 1P-5P-3N | 0.73 | 0.74 | 0.76 | 0.77 | 0.84 | 0.87 |
| 1P-5P-3N-4N | 0.56 | 0.59 | 0.62 | 0.64 | 0.84 | 0.87 |
| 1P-5P-2N-3N-9N | 0.25 | 0.30 | 0.34 | 0.39 | 0.84 | 0.87 |
| 1P-5P-2N-3N-4N-9N | 0.24 | 0.29 | 0.33 | 0.38 | 0.84 | 0.87 |
| 1P-5P-2N-3N-10N | 0.24 | 0.29 | 0.33 | 0.38 | 0.82 | 0.87 |
| 1P-5P-2N-3N-4N-10N | 0.24 | 0.29 | 0.33 | 0.38 | 0.82 | 0.87 |
| 1P-5P-2N-10N | 0.23 | 0.28 | 0.32 | 0.38 | 0.82 | 0.87 |
| 1P-5P-3N-10N | 0.23 | 0.28 | 0.32 | 0.38 | 0.82 | 0.87 |
| 1P-5P-2N-4N-10N | 0.22 | 0.28 | 0.32 | 0.37 | 0.82 | 0.87 |
| 1P-5P-2N-4N-9N | 0.21 | 0.27 | 0.31 | 0.36 | 0.83 | 0.87 |
| 1P-5P-2N-9N | 0.21 | 0.27 | 0.31 | 0.36 | 0.83 | 0.87 |
| 1P-5P-3N-9N | 0.21 | 0.26 | 0.29 | 0.34 | 0.82 | 0.87 |
| 1P-5P-3N-4N-10N | 0.18 | 0.23 | 0.29 | 0.35 | 0.82 | 0.87 |
| 1P-5P-3N-4N-9N | 0.15 | 0.21 | 0.24 | 0.29 | 0.81 | 0.87 |
| 1P-5P-10N | 0.05 | 0.15 | 0.21 | 0.28 | 0.80 | 0.87 |
| 1P-5P-4N-10N | 0.02 | 0.12 | 0.18 | 0.26 | 0.80 | 0.87 |
| 1P-5P-2N-3N-9N-10N | 0.08 | 0.10 | 0.13 | 0.17 | 0.79 | 0.87 |
| 1P-5P-2N-3N-4N-9N-10N | 0.08 | 0.10 | 0.13 | 0.17 | 0.79 | 0.87 |
| 1P-5P-2N-9N-10N | 0.08 | 0.10 | 0.13 | 0.17 | 0.79 | 0.87 |
| 1P-5P-2N-4N-9N-10N | 0.07 | 0.09 | 0.13 | 0.17 | 0.79 | 0.87 |
| 1P-5P-3N-9N-10N | 0.07 | 0.09 | 0.12 | 0.17 | 0.79 | 0.87 |
| 1P-5P-3N-4N-9N-10N | 0.05 | 0.08 | 0.12 | 0.15 | 0.79 | 0.87 |
| 1P-5P-9N-10N | 0.02 | 0.05 | 0.08 | 0.13 | 0.77 | 0.87 |
| 1P-5P-4N-9N-10N | 0.01 | 0.04 | 0.07 | 0.12 | 0.77 | 0.87 |
| 1P-5P-4N | 0.00 | 0.02 | 0.06 | 0.13 | 0.66 | 0.87 |
| 1P-5P | 0.00 | 0.01 | 0.04 | 0.10 | 0.61 | 0.87 |
| 1P-5P-4N-9N | 0.00 | 0.00 | 0.02 | 0.05 | 0.65 | 0.87 |
| 1P-5P-9N | 0.00 | 0.00 | 0.01 | 0.04 | 0.60 | 0.87 |
| 1P-5P-2N-3N-7N | 0.13 | 0.12 | 0.12 | 0.12 | 0.13 | 0.13 |
| 1P-5P-2N-3N-4N-7N | 0.12 | 0.12 | 0.12 | 0.12 | 0.13 | 0.13 |
| 1P-5P-2N-7N | 0.11 | 0.11 | 0.11 | 0.11 | 0.13 | 0.13 |
| 1P-5P-2N-4N-7N | 0.11 | 0.11 | 0.11 | 0.11 | 0.13 | 0.13 |
| 1P-5P-3N-7N | 0.11 | 0.10 | 0.10 | 0.10 | 0.12 | 0.13 |
| 1P-5P-2N-3N-8N | 0.10 | 0.10 | 0.10 | 0.10 | 0.10 | 0.10 |
| 1P-5P-2N-3N-4N-8N | 0.10 | 0.10 | 0.10 | 0.10 | 0.10 | 0.10 |
| 1P-5P-3N-4N-7N | 0.09 | 0.08 | 0.08 | 0.08 | 0.12 | 0.13 |
| 1P-5P-2N-8N | 0.09 | 0.09 | 0.09 | 0.09 | 0.10 | 0.10 |
| 1P-5P-2N-4N-8N | 0.09 | 0.09 | 0.09 | 0.09 | 0.10 | 0.10 |
| 1P-5P-3N-8N | 0.08 | 0.08 | 0.09 | 0.09 | 0.10 | 0.10 |
| 1P-5P-3N-4N-8N | 0.06 | 0.07 | 0.07 | 0.08 | 0.10 | 0.10 |
| 1P-5P-2N-3N-7N-9N | 0.04 | 0.05 | 0.05 | 0.06 | 0.12 | 0.13 |
| 1P-5P-2N-3N-4N-7N-9N | 0.04 | 0.04 | 0.05 | 0.06 | 0.12 | 0.13 |
| 1P-5P-2N-4N-7N-9N | 0.03 | 0.04 | 0.05 | 0.06 | 0.12 | 0.13 |
| 1P-5P-2N-7N-9N | 0.03 | 0.04 | 0.05 | 0.06 | 0.12 | 0.13 |
| 1P-5P-2N-3N-4N-7N-10N | 0.03 | 0.04 | 0.05 | 0.05 | 0.12 | 0.13 |
| 1P-5P-2N-3N-7N-10N | 0.03 | 0.04 | 0.05 | 0.05 | 0.12 | 0.13 |
| 1P-5P-3N-7N-9N | 0.04 | 0.04 | 0.04 | 0.05 | 0.12 | 0.13 |
| 1P-5P-2N-4N-7N-10N | 0.02 | 0.04 | 0.05 | 0.05 | 0.12 | 0.13 |
| 1P-5P-2N-7N-10N | 0.02 | 0.04 | 0.05 | 0.05 | 0.12 | 0.13 |
| 1P-5P-3N-7N-10N | 0.03 | 0.04 | 0.05 | 0.05 | 0.12 | 0.13 |
| 1P-5P-3N-4N-7N-10N | 0.02 | 0.03 | 0.04 | 0.05 | 0.12 | 0.13 |
| 1P-5P-3N-4N-7N-9N | 0.03 | 0.03 | 0.03 | 0.04 | 0.12 | 0.13 |
| 1P-5P-2N-3N-8N-10N | 0.03 | 0.03 | 0.04 | 0.05 | 0.10 | 0.10 |
| 1P-5P-2N-3N-4N-8N-10N | 0.03 | 0.03 | 0.04 | 0.05 | 0.10 | 0.10 |
| 1P-5P-2N-8N-10N | 0.03 | 0.03 | 0.04 | 0.05 | 0.10 | 0.10 |
| 1P-5P-3N-8N-10N | 0.03 | 0.03 | 0.04 | 0.05 | 0.10 | 0.10 |
| 1P-5P-2N-4N-8N-10N | 0.03 | 0.03 | 0.04 | 0.05 | 0.10 | 0.10 |
| 1P-5P-2N-3N-8N-9N | 0.02 | 0.03 | 0.04 | 0.04 | 0.10 | 0.10 |
| 1P-5P-2N-3N-4N-8N-9N | 0.02 | 0.03 | 0.04 | 0.04 | 0.10 | 0.10 |
| 1P-5P-2N-4N-8N-9N | 0.02 | 0.03 | 0.04 | 0.04 | 0.10 | 0.10 |
| 1P-5P-2N-8N-9N | 0.02 | 0.03 | 0.04 | 0.04 | 0.10 | 0.10 |
| 1P-5P-7N-10N | 0.00 | 0.02 | 0.03 | 0.04 | 0.11 | 0.13 |
| 1P-5P-3N-4N-8N-10N | 0.02 | 0.03 | 0.03 | 0.04 | 0.10 | 0.10 |
| 1P-5P-3N-8N-9N | 0.02 | 0.03 | 0.03 | 0.04 | 0.10 | 0.10 |
| 1P-5P-4N-7N-10N | 0.00 | 0.02 | 0.03 | 0.03 | 0.11 | 0.13 |
| 1P-5P-2N-3N-4N-7N-9N-10N | 0.01 | 0.02 | 0.02 | 0.03 | 0.11 | 0.13 |
| 1P-5P-2N-3N-7N-9N-10N | 0.01 | 0.02 | 0.02 | 0.03 | 0.11 | 0.13 |
| 1P-5P-2N-4N-7N-9N-10N | 0.01 | 0.01 | 0.02 | 0.03 | 0.11 | 0.13 |
| 1P-5P-2N-7N-9N-10N | 0.01 | 0.01 | 0.02 | 0.03 | 0.11 | 0.13 |
| 1P-5P-3N-7N-9N-10N | 0.01 | 0.01 | 0.02 | 0.03 | 0.11 | 0.13 |
| 1P-5P-3N-4N-8N-9N | 0.01 | 0.03 | 0.03 | 0.03 | 0.10 | 0.10 |
| 1P-5P-3N-4N-7N-9N-10N | 0.01 | 0.01 | 0.02 | 0.02 | 0.11 | 0.13 |
| 1P-5P-7N-9N-10N | 0.00 | 0.01 | 0.01 | 0.02 | 0.11 | 0.13 |
| 1P-5P-4N-7N-9N-10N | 0.00 | 0.01 | 0.01 | 0.02 | 0.11 | 0.13 |
| 1P-5P-8N-10N | 0.01 | 0.01 | 0.02 | 0.03 | 0.09 | 0.10 |
| 1P-5P-4N-8N-10N | 0.00 | 0.01 | 0.02 | 0.03 | 0.09 | 0.10 |
| 1P-5P-2N-3N-8N-9N-10N | 0.01 | 0.01 | 0.01 | 0.02 | 0.09 | 0.10 |
| 1P-5P-2N-8N-9N-10N | 0.01 | 0.01 | 0.01 | 0.02 | 0.09 | 0.10 |
| 1P-5P-2N-3N-4N-8N-9N-10N | 0.01 | 0.01 | 0.01 | 0.02 | 0.09 | 0.10 |
| 1P-5P-2N-4N-8N-9N-10N | 0.01 | 0.01 | 0.01 | 0.02 | 0.09 | 0.10 |
| 1P-5P-4N-7N | 0.00 | 0.00 | 0.00 | 0.01 | 0.10 | 0.13 |
| 1P-5P-7N | 0.00 | 0.00 | 0.00 | 0.01 | 0.10 | 0.13 |
| 1P-5P-3N-8N-9N-10N | 0.01 | 0.01 | 0.01 | 0.02 | 0.09 | 0.10 |
| 1P-5P-3N-4N-8N-9N-10N | 0.00 | 0.01 | 0.01 | 0.01 | 0.09 | 0.10 |
| 1P-5P-7N-9N | 0.00 | 0.00 | 0.00 | 0.00 | 0.10 | 0.13 |
| 1P-5P-4N-7N-9N | 0.00 | 0.00 | 0.00 | 0.00 | 0.10 | 0.13 |
| 1P-5P-4N-8N-9N-10N | 0.00 | 0.00 | 0.00 | 0.01 | 0.09 | 0.10 |
| 1P-5P-8N-9N-10N | 0.00 | 0.00 | 0.00 | 0.01 | 0.09 | 0.10 |
| 1P-5P-4N-8N | 0.00 | 0.00 | 0.01 | 0.01 | 0.08 | 0.10 |
| 1P-5P-8N | 0.00 | 0.00 | 0.00 | 0.01 | 0.07 | 0.10 |
| 1P-5P-4N-8N-9N | 0.00 | 0.00 | 0.00 | 0.01 | 0.08 | 0.10 |
| 1P-5P-8N-9N | 0.00 | 0.00 | 0.00 | 0.01 | 0.07 | 0.10 |

“Only_M” means only membrane-bound and “Only_D” means only diffusible. “D = 0.01M, 0.5M, M, 2M” means the ratios of diffusible to membrane-bound intercellular regulation are 0.01, 0.5, 1, and 2, respectively. Only topologies with *Q* ≥ 0.1 in at least one column are shown.
