# Supplementary material for: An Atlas of Network Topologies Reveals Design Principles for Caenorhabditis elegans Vulval Precursor Cell Fate Patterning
Source: PLoS One. 2015 Jun 26;10(6):e0131397. doi: 10.1371/journal.pone.0131397 (PMC4482679; doi:10.1371/journal.pone.0131397)
Supplement: S7 Table — (DOCX) [file pone.0131397.s013.docx]

**S7 Table. *Q* values of topologies for different ratios of diffusible to membrane-bound intercellular regulation for S2 = 0.1.**

|  | Only D | D = 2M | D = M | D = 0.5M | D = 0.01M | Only M |
| --- | --- | --- | --- | --- | --- | --- |
| 1P-2P-3N-6P | 0.14 | 0.18 | 0.20 | 0.23 | 0.45 | 0.55 |
| 1P-2P-3N-4N-6P-10N | 0.05 | 0.09 | 0.12 | 0.16 | 0.55 | 0.59 |
| 1P-2P-3N-6P-10N | 0.03 | 0.06 | 0.08 | 0.11 | 0.49 | 0.64 |
| 1P-2P-4N-6P-10N | 0.02 | 0.07 | 0.11 | 0.15 | 0.51 | 0.55 |
| 1P-2P-4N-10N | 0.02 | 0.08 | 0.11 | 0.15 | 0.47 | 0.51 |
| 1P-2P-3N-4N-6P-9N-10N | 0.01 | 0.04 | 0.06 | 0.09 | 0.52 | 0.57 |
| 1P-2P-3N-6P-9N | 0.04 | 0.07 | 0.10 | 0.14 | 0.43 | 0.45 |
| 1P-2P-3N-6P-9N-10N | 0.01 | 0.02 | 0.04 | 0.08 | 0.50 | 0.58 |
| 1P-2P-4N-6P-9N-10N | 0.00 | 0.03 | 0.05 | 0.08 | 0.50 | 0.55 |
| 1P-2P-4N-9N-10N | 0.00 | 0.03 | 0.05 | 0.08 | 0.47 | 0.52 |
| 1P-2P-6P-10N | 0.01 | 0.03 | 0.05 | 0.08 | 0.42 | 0.56 |
| 1P-5P-2N-4N | 0.15 | 0.16 | 0.16 | 0.17 | 0.23 | 0.23 |
| 1P-2P-6P-9N-10N | 0.00 | 0.01 | 0.02 | 0.05 | 0.45 | 0.56 |
| 1P-5P-3N-4N | 0.13 | 0.15 | 0.16 | 0.16 | 0.23 | 0.25 |
| 1P-2P-3N-4N-10N | 0.04 | 0.08 | 0.10 | 0.12 | 0.35 | 0.37 |
| 1P-2P-3N-4N-9N-10N | 0.01 | 0.03 | 0.05 | 0.07 | 0.37 | 0.40 |
| 1P-5P-3N | 0.14 | 0.15 | 0.15 | 0.15 | 0.16 | 0.16 |
| 1P-2P-5P-4N-10N | 0.01 | 0.05 | 0.06 | 0.09 | 0.27 | 0.30 |
| 1P-5P-3N-4N-9N | 0.04 | 0.06 | 0.07 | 0.08 | 0.26 | 0.28 |
| 1P-5P-3N-9N | 0.04 | 0.06 | 0.07 | 0.09 | 0.25 | 0.26 |
| 1P-2P-3N-4N-6P | 0.08 | 0.09 | 0.10 | 0.11 | 0.20 | 0.21 |
| 1P-5P-4N-10N | 0.01 | 0.04 | 0.06 | 0.08 | 0.27 | 0.29 |
| 1P-5P-3N-4N-10N | 0.04 | 0.06 | 0.08 | 0.10 | 0.24 | 0.25 |
| 1P-5P-2N-4N-10N | 0.05 | 0.07 | 0.08 | 0.09 | 0.23 | 0.24 |
| 1P-2P-5P-3N-4N | 0.09 | 0.10 | 0.11 | 0.11 | 0.16 | 0.17 |
| 1P-2P-3N | 0.12 | 0.12 | 0.12 | 0.12 | 0.12 | 0.12 |
| 1P-5P-2N-3N-4N | 0.11 | 0.11 | 0.11 | 0.11 | 0.12 | 0.12 |
| 1P-2P-5P-3N | 0.10 | 0.11 | 0.11 | 0.11 | 0.12 | 0.12 |
| 1P-5P-3N-4N-9N-10N | 0.01 | 0.02 | 0.03 | 0.05 | 0.26 | 0.28 |
| 1P-5P-4N-9N-10N | 0.00 | 0.01 | 0.03 | 0.04 | 0.27 | 0.29 |
| 1P-5P-3N-9N-10N | 0.01 | 0.02 | 0.03 | 0.05 | 0.24 | 0.27 |
| 1P-2P-5P-4N-9N-10N | 0.00 | 0.02 | 0.03 | 0.05 | 0.23 | 0.26 |
| 1P-5P-9N-10N | 0.00 | 0.01 | 0.02 | 0.04 | 0.25 | 0.27 |
| 1P-2P-5P-3N-4N-10N | 0.02 | 0.04 | 0.06 | 0.07 | 0.19 | 0.20 |
| 1P-5P-2N-4N-9N | 0.05 | 0.06 | 0.07 | 0.08 | 0.16 | 0.17 |
| 1P-2P-3N-9N | 0.04 | 0.05 | 0.06 | 0.07 | 0.17 | 0.18 |
| 1P-5P-4N | 0.00 | 0.01 | 0.02 | 0.05 | 0.21 | 0.28 |
| 1P-5P-3N-10N | 0.04 | 0.05 | 0.06 | 0.07 | 0.17 | 0.18 |
| 1P-5P-4N-9N | 0.00 | 0.00 | 0.01 | 0.02 | 0.22 | 0.29 |
| 1P-2P-5P-3N-4N-9N | 0.03 | 0.04 | 0.04 | 0.05 | 0.18 | 0.19 |
| 1P-2P-5P-3N-9N | 0.03 | 0.04 | 0.05 | 0.06 | 0.17 | 0.18 |
| 1P-2P-3N-4N-6P-9N | 0.03 | 0.04 | 0.05 | 0.06 | 0.17 | 0.17 |
| 1P-2P-5P-3N-4N-9N-10N | 0.01 | 0.02 | 0.03 | 0.04 | 0.20 | 0.22 |
| 1P-5P-10N | 0.01 | 0.03 | 0.04 | 0.06 | 0.17 | 0.19 |
| 1P-2P-3N-9N-10N | 0.01 | 0.02 | 0.03 | 0.04 | 0.19 | 0.21 |
| 1P-5P-9N | 0.00 | 0.00 | 0.01 | 0.02 | 0.19 | 0.27 |
| 1P-5P-2N-4N-9N-10N | 0.01 | 0.03 | 0.03 | 0.04 | 0.16 | 0.17 |
| 1P-2P-3N-10N | 0.03 | 0.04 | 0.04 | 0.06 | 0.13 | 0.14 |
| 1P-2P-5P-3N-9N-10N | 0.01 | 0.02 | 0.02 | 0.03 | 0.17 | 0.18 |
| 1P-2P-9N-10N | 0.00 | 0.01 | 0.02 | 0.03 | 0.17 | 0.19 |
| 1P-5P-2N-3N-4N-10N | 0.03 | 0.04 | 0.05 | 0.05 | 0.12 | 0.13 |
| 1P-2P-5P-9N-10N | 0.00 | 0.01 | 0.01 | 0.03 | 0.16 | 0.19 |
| 1P-5P-2N-3N-4N-9N | 0.03 | 0.04 | 0.04 | 0.05 | 0.12 | 0.12 |
| 1P-2P-5P-3N-10N | 0.02 | 0.03 | 0.04 | 0.05 | 0.11 | 0.12 |
| 1P-2P-5P-4N | 0.00 | 0.01 | 0.01 | 0.03 | 0.14 | 0.19 |
| 1P-2P-3N-4N-9N | 0.03 | 0.04 | 0.04 | 0.05 | 0.11 | 0.11 |
| 1P-2P-5P-4N-9N | 0.00 | 0.00 | 0.00 | 0.01 | 0.15 | 0.20 |
| 1P-2P-10N | 0.01 | 0.02 | 0.03 | 0.04 | 0.12 | 0.13 |
| 1P-2P-5P-10N | 0.01 | 0.02 | 0.03 | 0.04 | 0.12 | 0.13 |
| 1P-5P | 0.00 | 0.00 | 0.01 | 0.03 | 0.12 | 0.16 |
| 1P-2P-5P-9N | 0.00 | 0.00 | 0.00 | 0.01 | 0.12 | 0.18 |
| 1P-5P-2N-3N-4N-9N-10N | 0.01 | 0.02 | 0.02 | 0.03 | 0.11 | 0.12 |
| 2P-3N-6P-9N-10N | 0.00 | 0.00 | 0.00 | 0.00 | 0.10 | 0.17 |
| 2P-3N-4N-6P-9N-10N | 0.00 | 0.00 | 0.00 | 0.00 | 0.10 | 0.16 |
| 2P-4N-6P-9N-10N | 0.00 | 0.00 | 0.00 | 0.00 | 0.10 | 0.15 |
| 2P-6P-9N-10N | 0.00 | 0.00 | 0.00 | 0.00 | 0.09 | 0.15 |
| 2P-3N-6P-9N | 0.00 | 0.00 | 0.01 | 0.00 | 0.09 | 0.13 |
| 1P-2P-5P | 0.00 | 0.00 | 0.01 | 0.02 | 0.08 | 0.11 |

“Only_M” means only membrane-bound and “Only_D” means only diffusible. “D = 0.01M, 0.5M, M, 2M” means the ratios of diffusible to membrane-bound intercellular regulation are 0.01, 0.5, 1, and 2, respectively. Only topologies with *Q* ≥ 0.1 in at least one column are shown.
