# Supplementary material for: An Atlas of Network Topologies Reveals Design Principles for Caenorhabditis elegans Vulval Precursor Cell Fate Patterning
Source: PLoS One. 2015 Jun 26;10(6):e0131397. doi: 10.1371/journal.pone.0131397 (PMC4482679; doi:10.1371/journal.pone.0131397)
Supplement: S8 Table — (DOCX) [file pone.0131397.s014.docx]

**S8 Table. *Q* values of topologies for different ratios of diffusible to membrane-bound intercellular regulation for S2 = 0.5.**

|  | Only D | D = 2M | D = M | D = 0.5M | D = 0.01M | Only M |
| --- | --- | --- | --- | --- | --- | --- |
| 1P-2P-3N-4N-6P-10N | 0.04 | 0.73 | 0.20 | 0.11 | 0.14 | 0.80 |
| 1P-2P-4N-6P-10N | 0.03 | 0.69 | 0.20 | 0.10 | 0.14 | 0.75 |
| 1P-2P-3N-6P | 0.03 | 0.58 | 0.18 | 0.10 | 0.14 | 0.74 |
| 1P-2P-4N-10N | 0.03 | 0.62 | 0.19 | 0.10 | 0.14 | 0.67 |
| 1P-2P-3N-4N-6P-9N-10N | 0.01 | 0.70 | 0.11 | 0.05 | 0.07 | 0.77 |
| 1P-2P-3N-6P-10N | 0.01 | 0.63 | 0.09 | 0.04 | 0.05 | 0.86 |
| 1P-2P-4N-6P-9N-10N | 0.00 | 0.68 | 0.10 | 0.04 | 0.06 | 0.75 |
| 1P-2P-3N-6P-9N-10N | 0.00 | 0.64 | 0.08 | 0.02 | 0.04 | 0.75 |
| 1P-2P-4N-9N-10N | 0.00 | 0.63 | 0.10 | 0.04 | 0.06 | 0.70 |
| 1P-2P-3N-6P-9N | 0.01 | 0.54 | 0.14 | 0.05 | 0.09 | 0.58 |
| 1P-2P-6P-9N-10N | 0.00 | 0.58 | 0.04 | 0.01 | 0.02 | 0.76 |
| 1P-2P-6P-10N | 0.00 | 0.53 | 0.06 | 0.02 | 0.03 | 0.76 |
| 1P-2P-3N-4N-10N | 0.02 | 0.37 | 0.12 | 0.07 | 0.09 | 0.38 |
| 1P-2P-3N-4N-9N-10N | 0.01 | 0.38 | 0.06 | 0.03 | 0.04 | 0.41 |
| 1P-2P-3N-4N-6P | 0.05 | 0.26 | 0.11 | 0.06 | 0.08 | 0.28 |
| 1P-2P-5P-4N-10N | 0.01 | 0.24 | 0.10 | 0.05 | 0.07 | 0.26 |
| 1P-2P-3N-4N-6P-9N | 0.02 | 0.22 | 0.06 | 0.03 | 0.05 | 0.23 |
| 1P-2P-5P-4N-9N-10N | 0.00 | 0.17 | 0.05 | 0.02 | 0.04 | 0.19 |
| 2P-3N-6P-9N-10N | 0.00 | 0.14 | 0.00 | 0.00 | 0.00 | 0.23 |
| 2P-3N-4N-6P-9N-10N | 0.00 | 0.14 | 0.00 | 0.00 | 0.00 | 0.21 |
| 2P-6P-9N-10N | 0.00 | 0.12 | 0.00 | 0.00 | 0.00 | 0.20 |
| 2P-4N-6P-9N-10N | 0.00 | 0.12 | 0.00 | 0.00 | 0.00 | 0.20 |
| 2P-3N-6P-9N | 0.01 | 0.12 | 0.01 | 0.00 | 0.01 | 0.19 |
| 1P-2P-3N-4N-6P-7N-10N | 0.01 | 0.11 | 0.03 | 0.02 | 0.02 | 0.12 |
| 1P-2P-5P-3N-4N-10N | 0.01 | 0.10 | 0.04 | 0.02 | 0.03 | 0.11 |
| 1P-2P-3N-5N-6P | 0.01 | 0.08 | 0.04 | 0.02 | 0.03 | 0.10 |
| 1P-2P-4N-6P-7N-10N | 0.00 | 0.10 | 0.02 | 0.02 | 0.02 | 0.11 |
| 1P-2P-3N-4N-5N-6P-10N | 0.01 | 0.09 | 0.03 | 0.02 | 0.02 | 0.11 |
| 1P-2P-3N-6P-7N-10N | 0.00 | 0.10 | 0.02 | 0.01 | 0.01 | 0.13 |
| 1P-2P-3N-6P-7N | 0.00 | 0.09 | 0.03 | 0.02 | 0.02 | 0.11 |
| 1P-2P-5P-3N-4N-9N-10N | 0.00 | 0.10 | 0.02 | 0.01 | 0.02 | 0.11 |
| 1P-2P-4N-7N-10N | 0.00 | 0.09 | 0.02 | 0.02 | 0.02 | 0.10 |
| 1P-2P-3N-4N-6P-7N-9N-10N | 0.00 | 0.10 | 0.01 | 0.01 | 0.01 | 0.12 |
| 1P-2P-4N-6P-7N-9N-10N | 0.00 | 0.10 | 0.01 | 0.01 | 0.01 | 0.11 |
| 1P-2P-3N-6P-7N-9N-10N | 0.00 | 0.10 | 0.01 | 0.00 | 0.01 | 0.12 |
| 1P-2P-3N-5N-6P-10N | 0.00 | 0.08 | 0.02 | 0.01 | 0.01 | 0.11 |
| 1P-2P-4N-7N-9N-10N | 0.00 | 0.09 | 0.01 | 0.01 | 0.01 | 0.10 |
| 1P-2P-4N-5N-6P-9N-10N | 0.00 | 0.10 | 0.01 | 0.00 | 0.01 | 0.10 |
| 1P-2P-6P-7N-10N | 0.00 | 0.08 | 0.01 | 0.00 | 0.00 | 0.12 |
| 1P-2P-6P-7N-9N-10N | 0.00 | 0.08 | 0.00 | 0.00 | 0.00 | 0.12 |

“Only_M” means only membrane-bound and “Only_D” means only diffusible. “D = 0.01M, 0.5M, M, 2M” means the ratios of diffusible to membrane-bound intercellular regulation are 0.01, 0.5, 1, and 2, respectively. Only topologies with *Q* ≥ 0.1 in at least one column are shown.
