# Supplementary material for: An Atlas of Network Topologies Reveals Design Principles for Caenorhabditis elegans Vulval Precursor Cell Fate Patterning
Source: PLoS One. 2015 Jun 26;10(6):e0131397. doi: 10.1371/journal.pone.0131397 (PMC4482679; doi:10.1371/journal.pone.0131397)
Supplement: S9 Table — (DOCX) [file pone.0131397.s015.docx]

| Topology | S1 | 0.1 | 0.5 | 1 | 1 | 1 | 1 | 1 | 1 |
| --- | --- | --- | --- | --- | --- | --- | --- | --- | --- |
|  | S2 | 0 | 0 | 0 | 0.01 | 0.1 | 0.5 | 0.5 | 0.5 |
|  | S3 | 0 | 0 | 0 | 0 | 0 | 0 | 0.01 | 0.1 |
| 1P-5P-2N |  | 0.37 | 0.67 | 0.81 | 0.28 | 0.05 | 0.00 | 0.00 | 0.00 |
| 1P-5P-3N |  | 0.55 | 0.74 | 0.81 | 0.41 | 0.15 | 0.00 | 0.00 | 0.00 |
| 1P-5P-2N-3N |  | 0.58 | 0.79 | 0.88 | 0.30 | 0.05 | 0.00 | 0.00 | 0.00 |
| 1P-5P-2N-4N |  | 0.38 | 0.67 | 0.80 | 0.48 | 0.18 | 0.02 | 0.01 | 0.00 |
| 1P-5P-2N-3N-4N |  | 0.55 | 0.77 | 0.87 | 0.41 | 0.13 | 0.02 | 0.01 | 0.01 |
| 1P-2P-3N |  | 0.00 | 0.00 | 0.00 | 0.16 | 0.12 | 0.01 | 0.00 | 0.00 |
| 1P-2P-4N-10N |  | 0.00 | 0.00 | 0.00 | 0.08 | 0.17 | 0.22 | 0.11 | 0.04 |
| 1P-2P-3N-6P |  | 0.00 | 0.00 | 0.00 | 0.19 | 0.23 | 0.19 | 0.06 | 0.01 |
| 1P-2P-3N-6P-9N |  | 0.00 | 0.00 | 0.00 | 0.10 | 0.15 | 0.15 | 0.05 | 0.01 |
| 1P-2P-4N-6P-10N |  | 0.00 | 0.00 | 0.00 | 0.08 | 0.17 | 0.22 | 0.10 | 0.04 |
| 1P-2P-3N-4N-6P-10N |  | 0.00 | 0.00 | 0.00 | 0.10 | 0.18 | 0.22 | 0.10 | 0.04 |

**S9 Table. *Q* values of top topologies with different AC signal levels with “AND” rule.**

The topologies are the same as in S3 Table.
