# Supplementary material for: An Atlas of Network Topologies Reveals Design Principles for Caenorhabditis elegans Vulval Precursor Cell Fate Patterning
Source: PLoS One. 2015 Jun 26;10(6):e0131397. doi: 10.1371/journal.pone.0131397 (PMC4482679; doi:10.1371/journal.pone.0131397)
Supplement: S10 Table — (DOCX) [file pone.0131397.s016.docx]

| Topology | S1 | 0.1 | 0.5 | 1 | 1 | 1 | 1 | 1 | 1 |
| --- | --- | --- | --- | --- | --- | --- | --- | --- | --- |
|  | S2 | 0 | 0 | 0 | 0.01 | 0.1 | 0.5 | 0.5 | 0.5 |
|  | S3 | 0 | 0 | 0 | 0 | 0 | 0 | 0.01 | 0.1 |
| 1P-5P-2N-3N-7P-8P |  | 0.70 | 0.88 | 0.91 | 0.31 | 0.06 | 0.00 | 0.00 | 0.00 |
| 1P-5P-2N-3N-4N-7P-8P |  | 0.65 | 0.85 | 0.90 | 0.39 | 0.09 | 0.01 | 0.01 | 0.00 |
| 1P-5P-3N-7P-8P |  | 0.68 | 0.84 | 0.88 | 0.42 | 0.14 | 0.00 | 0.00 | 0.00 |
| 1P-5P-2N-3N-8P |  | 0.57 | 0.79 | 0.87 | 0.32 | 0.06 | 0.00 | 0.00 | 0.00 |
| 1P-5P-2N-3N-4N-8P |  | 0.53 | 0.77 | 0.86 | 0.39 | 0.09 | 0.01 | 0.01 | 0.01 |
| 1P-5P-2N-4N-8P |  | 0.37 | 0.65 | 0.78 | 0.39 | 0.11 | 0.01 | 0.01 | 0.00 |
| 1P-5P-2N-4N-7P-8P |  | 0.42 | 0.70 | 0.81 | 0.39 | 0.10 | 0.01 | 0.00 | 0.00 |
| 1P-5P-2N-3P-4N-7P-8P |  | 0.36 | 0.66 | 0.77 | 0.40 | 0.11 | 0.01 | 0.00 | 0.00 |
| 1P-2P-5P-3N-7P-8P |  | 0.67 | 0.83 | 0.87 | 0.44 | 0.15 | 0.00 | 0.00 | 0.00 |
| 1P-5P-3N-4N-7P-8P |  | 0.55 | 0.69 | 0.72 | 0.41 | 0.17 | 0.02 | 0.02 | 0.02 |
| 1P-2P-5P-3N-8P |  | 0.51 | 0.69 | 0.75 | 0.41 | 0.15 | 0.01 | 0.00 | 0.00 |
| 1P-5P-3N-8P |  | 0.53 | 0.71 | 0.78 | 0.40 | 0.14 | 0.00 | 0.00 | 0.00 |
| 1P-2P-5P-3P-4N-8P-10N |  | 0.06 | 0.06 | 0.06 | 0.15 | 0.24 | 0.28 | 0.14 | 0.05 |
| 1P-2P-5P-3P-4N-7P-8P-10N |  | 0.07 | 0.07 | 0.07 | 0.16 | 0.24 | 0.28 | 0.13 | 0.04 |
| 1P-2P-5P-4N-8N-10N |  | 0.10 | 0.08 | 0.08 | 0.16 | 0.23 | 0.29 | 0.14 | 0.05 |
| 1P-2P-5P-4N-7P-8P-10N |  | 0.12 | 0.10 | 0.10 | 0.16 | 0.23 | 0.29 | 0.13 | 0.04 |
| 1P-2P-3P-4N-8P-10N |  | 0.00 | 0.00 | 0.00 | 0.13 | 0.23 | 0.28 | 0.14 | 0.04 |
| 1P-2P-3P-4N-7P-8P-10N |  | 0.00 | 0.00 | 0.00 | 0.13 | 0.23 | 0.28 | 0.13 | 0.04 |
| 1P-2P-4N-8P-10N |  | 0.00 | 0.00 | 0.00 | 0.12 | 0.23 | 0.29 | 0.14 | 0.05 |
| 1P-2P-4N-7P-8P-10N |  | 0.00 | 0.00 | 0.00 | 0.12 | 0.23 | 0.29 | 0.13 | 0.04 |
| 1P-2P-5P-3N-4N-8P-10N |  | 0.18 | 0.20 | 0.22 | 0.20 | 0.19 | 0.18 | 0.09 | 0.04 |

**S10 Table. *Q* values of top topologies with different AC signal levels with “Combined AND & Additive” rule.**

The topologies are the same as in S4 Table.
