# Supplementary material for: An Atlas of Network Topologies Reveals Design Principles for Caenorhabditis elegans Vulval Precursor Cell Fate Patterning
Source: PLoS One. 2015 Jun 26;10(6):e0131397. doi: 10.1371/journal.pone.0131397 (PMC4482679; doi:10.1371/journal.pone.0131397)
Supplement: S11 Table — (DOCX) [file pone.0131397.s017.docx]

**S11 Table. Topologies with frequencies of expected error patterns under simulated mutant AC signaling with “AND” rule.**

| Mutation | Error pattern | | | |
| --- | --- | --- | --- | --- |
| AC ablation | #1 | 3_2_2_2_3 | #2 | 3_2_3_2_3 |
|  | #3 | 3_3_1_3_3 | #4 | 3_3_2_3_3 |
|  | #5 | 3_3_3_3_3 |  |  |
| EGF overexpression | #6 | 1_1_1_1_1 | #7 | 1_2_1_2_1 |
|  | #8 | 2_1_1_1_2 | #9 | 2_2_1_2_2 |

| Topology | AC ablation  (ablation time = 100 min) | | | | | EGF overexpression  (S1=1, S2=0.5, S3=0.1) | | | |
| --- | --- | --- | --- | --- | --- | --- | --- | --- | --- |
|  | #1 | #2 | #3 | #4 | #5 | #6 | #7 | #8 | #9 |
| 1P-5P-2N-4N-10N | 0.001 | 0.596 | 0 | 0 | 0.128 | 0.331 | 0.017 | 0.072 | 0.001 |
| 1P-5P-3N-4N | 0.074 | 0.569 | 0 | 0.003 | 0.132 | 0.439 | 0 | 0.176 | 0.001 |
| 1P-2P-4N-6P-10N | 0 | 0 | 0.003 | 0.719 | 0.156 | 0.001 | 0.363 | 0 | 0 |
| 1P-5P-2N-3N | 0.173 | 0.407 | 0 | 0 | 0.156 | 0.538 | 0 | 0.07 | 0.002 |
| 1P-5P-2N | 0.413 | 0.024 | 0.003 | 0 | 0.022 | 0.339 | 0 | 0.067 | 0.001 |
| 1P-5P-3N-4N-10N | 0.002 | 0.562 | 0 | 0 | 0.131 | 0.41 | 0 | 0.083 | 0 |
| 1P-2P-3N-6P | 0 | 0 | 0 | 0 | 1 | 0 | 0.115 | 0.026 | 0.052 |
| 1P-5P-2N-3N-4N-7N | 0.231 | 0.431 | 0 | 0 | 0.107 | 0.336 | 0.002 | 0.066 | 0.001 |
| 1P-5P-2N-3N-7N | 0.235 | 0.452 | 0 | 0 | 0.099 | 0.376 | 0 | 0.027 | 0 |
| 1P-2P-5P-3N | 0.013 | 0.001 | 0.011 | 0 | 0.121 | 0.378 | 0 | 0.249 | 0.009 |
| 1P-5P-2N-4N-7N | 0.485 | 0.192 | 0 | 0 | 0.111 | 0.236 | 0.003 | 0.078 | 0.01 |
| 1P-2P-5P-3N-4N | 0.002 | 0.003 | 0.013 | 0 | 0.115 | 0.341 | 0.002 | 0.203 | 0.005 |
| 1P-5P-2N-3N-4N-10N | 0 | 0 | 0 | 0 | 1 | 0.504 | 0.006 | 0.059 | 0.001 |
| 1P-5P-2N-10N | 0.004 | 0.542 | 0 | 0 | 0.136 | 0.447 | 0 | 0.028 | 0 |
| 1P-5P-2N-3N-10N | 0 | 0.002 | 0.006 | 0 | 0.124 | 0.577 | 0 | 0.026 | 0.001 |
| 1P-5P-3N-10N | 0 | 0.002 | 0.006 | 0 | 0.127 | 0.58 | 0 | 0.079 | 0.002 |
| 1P-2P-3N-4N-6P | 0 | 0 | 0 | 0 | 1 | 0 | 0.153 | 0.011 | 0.101 |
| 1P-2P-3N-6P-9N | 0 | 0 | 0 | 0 | 1 | 0 | 0.065 | 0 | 0.11 |
| 1P-5P-3N | 0.133 | 0.451 | 0 | 0 | 0.161 | 0.539 | 0 | 0.188 | 0.01 |
| 1P-5P-2N-4N | 0.464 | 0.029 | 0.004 | 0 | 0.07 | 0.308 | 0.004 | 0.148 | 0.011 |
| 1P-2P-4N-10N | 0 | 0 | 0.286 | 0 | 0.393 | 0 | 0.408 | 0 | 0.002 |
| 1P-5P-2N-3N-4N | 0.006 | 0.005 | 0.009 | 0 | 0.181 | 0.46 | 0.003 | 0.126 | 0.001 |
| 1P-2P-3N-4N-10N | 0.001 | 0.596 | 0 | 0 | 0.128 | 0.031 | 0.161 | 0.115 | 0.01 |
| 1P-2P-3N | 0.035 | 0.066 | 0.095 | 0 | 0.13 | 0.385 | 0 | 0.246 | 0.032 |

Only robust topologies with *Q* ≥ 0.1 for at least one S2 and the sum of frequencies ≥ 0.1 are shown.
