# Supplementary material for: An Atlas of Network Topologies Reveals Design Principles for Caenorhabditis elegans Vulval Precursor Cell Fate Patterning
Source: PLoS One. 2015 Jun 26;10(6):e0131397. doi: 10.1371/journal.pone.0131397 (PMC4482679; doi:10.1371/journal.pone.0131397)
Supplement: S12 Table — (DOCX) [file pone.0131397.s018.docx]

**S12 Table. Topologies with frequencies of expected error patterns under simulated mutant AC signaling with “Combined AND & Additive” rule.**

| Mutation | Error patterns | | | |
| --- | --- | --- | --- | --- |
| AC ablation | #1 | 3_2_2_2_3 | #2 | 3_2_3_2_3 |
|  | #3 | 3_3_1_3_3 | #4 | 3_3_2_3_3 |
|  | #5 | 3_3_3_3_3 |  |  |
| EGF overexpression | #6 | 1_1_1_1_1 | #7 | 1_2_1_2_1 |
|  | #8 | 2_1_1_1_2 | #9 | 2_2_1_2_2 |

| Topology | AC ablation  (ablation time = 100 min) | | | | | EGF overexpression  (S1=1, S2=0.5, S3=0.1) | | | |
| --- | --- | --- | --- | --- | --- | --- | --- | --- | --- |
|  | #1 | #2 | #3 | #4 | #5 | #6 | #7 | #8 | #9 |
| 1P-5P-3N-8P-10N | 0.002 | 0.553 | 0 | 0 | 0.14 | 0.556 | 0 | 0.074 | 0 |
| 1P-5P-3N-4N | 0 | 0 | 0 | 0 | 1 | 0.45 | 0 | 0.167 | 0.001 |
| 1P-5P-2N-4N-7P-8P | 0.464 | 0.029 | 0.004 | 0 | 0.07 | 0.413 | 0.002 | 0.178 | 0.032 |
| 1P-5P-2N-4N-7N-8P | 0.485 | 0.192 | 0 | 0 | 0.111 | 0.228 | 0.003 | 0.086 | 0.02 |
| 1P-2P-3N-4N-8P-10N | 0.001 | 0.596 | 0 | 0 | 0.128 | 0.035 | 0.335 | 0.005 | 0.008 |
| 1P-5P-2N-4N-7P-8P-10N | 0.003 | 0.088 | 0.001 | 0 | 0.03 | 0.417 | 0.056 | 0.095 | 0.003 |
| 1P-5P-2N-3N-7N-8P | 0.235 | 0.452 | 0 | 0 | 0.099 | 0.335 | 0 | 0.027 | 0.001 |
| 1P-5P-2N-3N | 0 | 0 | 0 | 0 | 1 | 0.534 | 0 | 0.078 | 0.003 |
| 1P-5P-2N-4N-7P-8P-9P | 0.54 | 0.033 | 0.001 | 0 | 0.115 | 0.551 | 0.004 | 0.174 | 0.023 |
| 1P-5P-2N-3N-4N-8P-10N | 0.003 | 0.543 | 0 | 0 | 0.141 | 0.516 | 0.028 | 0.062 | 0.001 |
| 1P-5P-3P-4N-7P-8P-10N | 0 | 0.209 | 0 | 0.001 | 0.026 | 0.033 | 0.145 | 0 | 0 |
| 1P-2P-5P-3N-10N | 0 | 0 | 0 | 0 | 1 | 0.576 | 0.003 | 0.083 | 0.002 |
| 1P-5P-2N-4N-10N | 0 | 0 | 0 | 0 | 1 | 0.372 | 0.032 | 0.077 | 0 |
| 1P-5P-3N-4N-9P | 0 | 0.001 | 0 | 0 | 0.719 | 0.456 | 0 | 0.151 | 0 |
| 1P-2P-5P-3N-8P-9N | 0.211 | 0.446 | 0 | 0.004 | 0.101 | 0.076 | 0 | 0.015 | 0.042 |
| 1P-2P-4N-7P-8P-10N | 0.002 | 0.233 | 0.03 | 0 | 0.062 | 0.003 | 0.527 | 0 | 0.001 |
| 1P-2P-5P-3N-4N | 0 | 0 | 0 | 0 | 1 | 0.579 | 0.002 | 0.188 | 0.042 |
| 1P-2P-4N-7P-10N | 0 | 0 | 0.286 | 0 | 0.393 | 0.006 | 0.485 | 0 | 0 |
| 1P-5P-2N-3P-4N-10N | 0 | 0 | 0 | 0 | 1 | 0.382 | 0.031 | 0.066 | 0 |
| 1P-5P-2N-3N-4N-7P | 0.006 | 0.005 | 0.009 | 0 | 0.181 | 0.579 | 0.007 | 0.136 | 0.01 |
| 1P-5P-2N-4N-8P-9P-10N | 0.005 | 0.394 | 0 | 0 | 0.089 | 0.479 | 0.047 | 0.088 | 0.004 |
| 1P-5P-2N-3N-4N-7P-8P-10N | 0.001 | 0.078 | 0 | 0 | 0.021 | 0.608 | 0.025 | 0.07 | 0.001 |
| 1P-2P-3N-4N-8P | 0.074 | 0.569 | 0 | 0.003 | 0.132 | 0.419 | 0 | 0.319 | 0.098 |
| 1P-5P-3N-4N-8P-9P-10N | 0 | 0.461 | 0 | 0 | 0.079 | 0.372 | 0.004 | 0.043 | 0.001 |
| 1P-5P-2N-3P-4N-8P-9P-10N | 0.007 | 0.367 | 0 | 0.01 | 0.076 | 0.491 | 0.035 | 0.078 | 0 |
| 1P-5P-2N-10N | 0 | 0 | 0 | 0 | 1 | 0.479 | 0.013 | 0.032 | 0.002 |
| 1P-5P-2N-3P-4N-8P | 0.569 | 0.016 | 0 | 0.027 | 0.133 | 0.351 | 0 | 0.158 | 0.015 |
| 1P-2N-3P-4N-7P-8P | 0.913 | 0 | 0 | 0.025 | 0.017 | 0.461 | 0 | 0.282 | 0.035 |
| 1P-2P-5P-3N-4N-8P-10N | 0.002 | 0.547 | 0 | 0.002 | 0.129 | 0.494 | 0.061 | 0.082 | 0.002 |
| 1P-5P-2N-3P-10N | 0 | 0 | 0 | 0 | 1 | 0.455 | 0.021 | 0.044 | 0.002 |
| 1P-5P-3N-7P-8P | 0.034 | 0.067 | 0.003 | 0 | 0.02 | 0.667 | 0 | 0.183 | 0.009 |
| 1P-2P-4N-9P-10N | 0 | 0 | 0 | 0 | 0.778 | 0.004 | 0.419 | 0 | 0 |
| 1P-5P-2N-3N-7P-8P | 0.06 | 0.062 | 0.001 | 0 | 0.024 | 0.667 | 0 | 0.08 | 0.002 |
| 1P-5P-2N-3N-4N-8P-9N | 0.18 | 0.463 | 0 | 0 | 0.123 | 0.067 | 0.005 | 0.007 | 0.028 |
| 1P-5P-2N-3N-4N-7P-9P | 0.002 | 0.006 | 0.002 | 0 | 0.146 | 0.675 | 0.005 | 0.159 | 0.014 |
| 1P-2P-5P-3P-4N-10N | 0 | 0 | 0 | 0 | 1 | 0.002 | 0.371 | 0 | 0 |
| 1P-2P-3P-4N-7P-10N | 0 | 0 | 0.019 | 0.002 | 0.76 | 0.004 | 0.458 | 0 | 0.001 |
| 1P-5P-3N-4N-7P-8P-10N | 0 | 0.089 | 0 | 0 | 0.021 | 0.485 | 0.004 | 0.079 | 0 |
| 1P-5P-3N | 0 | 0 | 0 | 0 | 1 | 0.557 | 0 | 0.165 | 0.012 |
| 1P-5P-2N-3N-4N-8P-9P | 0.194 | 0.316 | 0 | 0 | 0.096 | 0.591 | 0.001 | 0.167 | 0.03 |
| 1P-5P-2N-4N | 0 | 0 | 0 | 0 | 1 | 0.352 | 0.005 | 0.13 | 0.016 |
| 1P-2P-5P-3N-8P-10N | 0.003 | 0.533 | 0 | 0.024 | 0.133 | 0.562 | 0.005 | 0.09 | 0 |
| 1P-5P-2N-3N-4N-7P-10N | 0 | 0.003 | 0.004 | 0 | 0.128 | 0.621 | 0.014 | 0.065 | 0.001 |
| 1P-2P-4N-8P-10N | 0.002 | 0.591 | 0 | 0 | 0.122 | 0 | 0.459 | 0 | 0.001 |
| 1P-2P-3N-8P-10N | 0.002 | 0.593 | 0 | 0.018 | 0.12 | 0.344 | 0 | 0.124 | 0.004 |
| 1P-2P-3N-4N-7P-8P-9P-10N | 0.004 | 0.303 | 0 | 0 | 0.06 | 0.042 | 0.411 | 0 | 0.006 |
| 1P-2P-3P-4N-7P-8P-10N | 0 | 0.245 | 0.002 | 0.003 | 0.093 | 0.001 | 0.486 | 0.002 | 0 |
| 1P-2P-4N-7P-8P-9P-10N | 0.001 | 0.293 | 0.001 | 0 | 0.062 | 0.002 | 0.538 | 0 | 0 |
| 1P-2P-5P-3N-4N-8P-9P | 0.098 | 0.396 | 0 | 0.002 | 0.084 | 0.613 | 0.002 | 0.22 | 0.055 |
| 1P-2P-5P-3P-4N-8P-10N | 0.001 | 0.558 | 0 | 0 | 0.129 | 0.001 | 0.389 | 0 | 0 |
| 1P-2P-5P-3P-4N-8P-9P-10N | 0.001 | 0.462 | 0 | 0 | 0.098 | 0.001 | 0.404 | 0 | 0 |
| 1P-2P-5P-3N-4N-8P-9N | 0.108 | 0.521 | 0 | 0.002 | 0.134 | 0.087 | 0.026 | 0.012 | 0.072 |
| 1P-5P-2N-3P | 0 | 0 | 0 | 0 | 1 | 0.387 | 0 | 0.187 | 0.017 |
| 1P-5P-2N-3P-4N-7P | 0.026 | 0.007 | 0.001 | 0.008 | 0.769 | 0.437 | 0 | 0.156 | 0.007 |
| 1P-2P-5P-3N-7P-8P | 0.044 | 0.057 | 0.003 | 0.004 | 0.019 | 0.667 | 0 | 0.189 | 0.021 |
| 1P-2P-3P-4N-10N | 0 | 0 | 0 | 0 | 1 | 0.003 | 0.397 | 0 | 0.001 |
| 1P-2P-3N | 0 | 0 | 0 | 0 | 1 | 0.366 | 0 | 0.256 | 0.043 |
| 1P-5P-2N-3P-4N-7P-9P | 0.011 | 0.005 | 0.001 | 0 | 0.88 | 0.553 | 0 | 0.151 | 0.005 |
| 1P-5P-2N-8P-10N | 0.004 | 0.542 | 0 | 0 | 0.136 | 0.428 | 0.024 | 0.057 | 0.004 |
| 1P-5P-2N-3P-4N-8P-10N | 0.007 | 0.508 | 0 | 0.025 | 0.112 | 0.353 | 0.037 | 0.082 | 0 |
| 1P-5P-2N-3N-7P | 0.024 | 0.001 | 0.009 | 0 | 0.133 | 0.657 | 0 | 0.085 | 0.001 |
| 1P-2P-5P-3P-4N-7P-9P-10N | 0 | 0.002 | 0.002 | 0.001 | 0.548 | 0.002 | 0.404 | 0 | 0.001 |
| 1P-2P-5P-3N-4N-10N | 0 | 0 | 0 | 0 | 1 | 0.281 | 0.072 | 0.078 | 0.002 |
| 1P-2P-5P-3P-8P-10N | 0.003 | 0.575 | 0 | 0.014 | 0.114 | 0.033 | 0.229 | 0 | 0 |
| 1P-2P-5P-3N-4N-7P | 0.002 | 0.003 | 0.013 | 0 | 0.115 | 0.605 | 0.002 | 0.226 | 0.036 |
| 1P-5P-2N-8P | 0.392 | 0.193 | 0 | 0 | 0.162 | 0.356 | 0 | 0.174 | 0.021 |
| 1P-2P-3N-4N-7P-10N | 0 | 0 | 0.376 | 0 | 0.403 | 0.042 | 0.193 | 0.141 | 0.011 |
| 1P-5P-2N-3N-4N-9P | 0 | 0.001 | 0 | 0 | 0.708 | 0.595 | 0.005 | 0.157 | 0.016 |
| 1P-5P-2N-3P-4N-7P-8P-10N | 0.007 | 0.09 | 0.001 | 0.025 | 0.036 | 0.396 | 0.049 | 0.101 | 0.002 |
| 1P-2P-3N-4N-7P | 0 | 0 | 0.643 | 0 | 0.115 | 0.507 | 0 | 0.354 | 0.078 |
| 1P-2P-5P-3N-4N-7P-8P-9P-10N | 0.003 | 0.295 | 0 | 0 | 0.065 | 0.038 | 0.382 | 0.001 | 0.002 |
| 1P-5P-3N-4N-7P-8P-9P-10N | 0 | 0.111 | 0 | 0 | 0.022 | 0.391 | 0.011 | 0.049 | 0.001 |
| 1P-2P-5P-3N-4N-7P-10N | 0 | 0.002 | 0.013 | 0 | 0.231 | 0.353 | 0.077 | 0.118 | 0.001 |
| 1P-2P-5P-7P-10N | 0 | 0.002 | 0.002 | 0 | 0.121 | 0.392 | 0.075 | 0.017 | 0 |
| 1P-5P-2N-3N-10N | 0 | 0 | 0 | 0 | 1 | 0.583 | 0 | 0.036 | 0 |
| 1P-5P-3N-4N-9P-10N | 0 | 0.001 | 0 | 0 | 0.723 | 0.354 | 0.002 | 0.05 | 0 |
| 1P-5P-3N-10N | 0 | 0 | 0 | 0 | 1 | 0.567 | 0 | 0.085 | 0 |
| 1P-5P-2N-3P-7P-8P | 0.626 | 0.001 | 0 | 0.024 | 0.014 | 0.512 | 0 | 0.168 | 0.015 |
| 1P-5P-2N-4N-7P-9P | 0.011 | 0.023 | 0.004 | 0 | 0.687 | 0.551 | 0.007 | 0.152 | 0.018 |
| 1P-2P-5P-3P-4N-7P-8P-9N-10N | 0 | 0.243 | 0 | 0 | 0.059 | 0.001 | 0.219 | 0 | 0.002 |
| 1P-2P-5P-3P-4N-7P-8P-9P-10N | 0.001 | 0.265 | 0 | 0.001 | 0.067 | 0.001 | 0.446 | 0 | 0 |
| 1P-5P-2N-7P-8P | 0.413 | 0.024 | 0.003 | 0 | 0.022 | 0.517 | 0 | 0.154 | 0.014 |
| 1P-2P-3P-4N-7P-8P-9P-10N | 0 | 0.303 | 0 | 0 | 0.071 | 0.001 | 0.478 | 0 | 0 |
| 1P-5P-2N-3N-4N-9N-10N | 0 | 0 | 0 | 0 | 1 | 0.082 | 0.017 | 0.005 | 0 |
| 1P-5P-3N-4N-8P | 0.081 | 0.526 | 0 | 0 | 0.157 | 0.459 | 0 | 0.173 | 0.01 |
| 1P-5P-2N-3N-4N | 0 | 0 | 0 | 0 | 1 | 0.479 | 0.009 | 0.114 | 0.013 |
| 1P-5P-3P-10N | 0 | 0 | 0 | 0 | 1 | 0.05 | 0.075 | 0 | 0 |
| 1P-5P-2N-4N-9P-10N | 0 | 0.004 | 0.001 | 0 | 0.695 | 0.491 | 0.035 | 0.082 | 0.001 |
| 1P-5P-2N-3N-4N-8P-9P-10N | 0.003 | 0.394 | 0 | 0 | 0.068 | 0.668 | 0.024 | 0.071 | 0.003 |
| 1P-2N-3P-4N-8P-9P | 0.851 | 0.001 | 0 | 0.071 | 0.027 | 0.529 | 0 | 0.149 | 0.018 |
| 1P-5P-2N-3N-8P-10N | 0.003 | 0.551 | 0 | 0 | 0.137 | 0.576 | 0 | 0.037 | 0 |
| 1P-5P-2N-4N-8P-10N | 0.002 | 0.544 | 0 | 0 | 0.134 | 0.347 | 0.044 | 0.092 | 0.005 |
| 1P-5P-2N-3P-4N-7P-8P | 0.594 | 0.002 | 0 | 0.023 | 0.11 | 0.42 | 0 | 0.181 | 0.016 |
| 1P-5P-2N-3N-4N-8P | 0.196 | 0.402 | 0 | 0 | 0.152 | 0.467 | 0.002 | 0.124 | 0.028 |
| 1P-2N-3P-4N-7P-8P-9P | 0.903 | 0 | 0 | 0.022 | 0.031 | 0.599 | 0 | 0.164 | 0.015 |
| 1P-5P-2N-4N-8P | 0.396 | 0.194 | 0 | 0 | 0.163 | 0.342 | 0.001 | 0.162 | 0.031 |
| 1P-2P-5P-4N-7P-8P-10N | 0.001 | 0.203 | 0.001 | 0 | 0.063 | 0.001 | 0.418 | 0 | 0 |
| 1P-5P-3N-4N-7P-10N | 0 | 0.003 | 0.005 | 0 | 0.151 | 0.479 | 0.001 | 0.085 | 0 |
| 1P-5P-2N-3N-4N-7P-8P-9P | 0.091 | 0.042 | 0.001 | 0 | 0.023 | 0.671 | 0.004 | 0.168 | 0.033 |
| 1P-2P-3N-4N-7P-8P | 0.012 | 0.07 | 0.087 | 0 | 0.015 | 0.485 | 0 | 0.376 | 0.083 |
| 1P-5P-2N-3P-8P | 0.571 | 0.015 | 0 | 0.031 | 0.133 | 0.368 | 0 | 0.196 | 0.02 |
| 1P-5P-3P-7P-8P-10N | 0 | 0.08 | 0.001 | 0.008 | 0.014 | 0.407 | 0.064 | 0.004 | 0 |
| 1P-2P-5P-4N-7P-10N | 0 | 0.001 | 0.024 | 0 | 0.418 | 0.002 | 0.386 | 0 | 0 |
| 1P-2P-5P-3N-7P | 0.013 | 0.001 | 0.011 | 0 | 0.121 | 0.666 | 0 | 0.213 | 0.013 |
| 1P-5P-2N-3P-4N-9P | 0 | 0 | 0 | 0 | 0.98 | 0.484 | 0 | 0.151 | 0.003 |
| 1P-5P-2N-3N-4N-9P-10N | 0 | 0.001 | 0 | 0 | 0.63 | 0.644 | 0.027 | 0.073 | 0 |
| 1P-5P-2N-3P-4N-7N-8P | 0.665 | 0.016 | 0 | 0.011 | 0.098 | 0.234 | 0 | 0.086 | 0.012 |
| 1P-2P-3P-4N-7P-8P-9N-10N | 0 | 0.267 | 0.004 | 0.003 | 0.075 | 0.001 | 0.249 | 0 | 0 |
| 1P-5P-3N-4N-8P-9P | 0.074 | 0.456 | 0 | 0 | 0.092 | 0.468 | 0 | 0.167 | 0.005 |
| 1P-2P-5P-3N-9N | 0 | 0 | 0 | 0 | 1 | 0.075 | 0 | 0.013 | 0.035 |
| 1P-2P-5P-3N | 0 | 0 | 0 | 0 | 1 | 0.568 | 0 | 0.184 | 0.022 |
| 1P-2P-5P-3N-4N-7P-9N | 0.002 | 0.003 | 0.007 | 0 | 0.118 | 0.05 | 0.043 | 0.001 | 0.062 |
| 1P-5P-2N-7P | 0.222 | 0 | 0.009 | 0 | 0.135 | 0.564 | 0 | 0.123 | 0.011 |
| 1P-5P-3N-4N-10N | 0 | 0 | 0 | 0 | 1 | 0.397 | 0 | 0.062 | 0 |
| 1P-2P-4N-10N | 0 | 0 | 0 | 0 | 1 | 0.005 | 0.407 | 0 | 0 |
| 1P-2P-5P-4N-8P-10N | 0.001 | 0.548 | 0 | 0 | 0.139 | 0 | 0.369 | 0 | 0 |
| 1P-2P-5P-10N | 0 | 0 | 0 | 0 | 1 | 0.028 | 0.173 | 0 | 0.001 |
| 1P-2P-3N-7P | 0 | 0 | 0.622 | 0 | 0.13 | 0.523 | 0 | 0.283 | 0.023 |
| 1P-5P-2N-3P-4N-7P-10N | 0 | 0.013 | 0.001 | 0.006 | 0.307 | 0.44 | 0.038 | 0.086 | 0.001 |
| 1P-2P-3P-4N-8P-9P-10N | 0 | 0.518 | 0 | 0 | 0.093 | 0.001 | 0.425 | 0 | 0 |
| 1P-3P-4N-8P-10N | 0 | 0.916 | 0 | 0.001 | 0.015 | 0.008 | 0.467 | 0 | 0.001 |
| 1P-5P-3P-4N-7P-10N | 0.001 | 0.004 | 0 | 0 | 0.226 | 0.039 | 0.11 | 0 | 0.001 |
| 1P-2P-5P-4N-8P-9P-10N | 0.001 | 0.46 | 0 | 0 | 0.106 | 0 | 0.383 | 0 | 0 |
| 1P-5P-2N-3N-7P-10N | 0 | 0.002 | 0.006 | 0 | 0.124 | 0.695 | 0 | 0.038 | 0 |
| 1P-3P-4N-7P-8P-10N | 0 | 0.331 | 0 | 0.002 | 0.013 | 0.013 | 0.521 | 0 | 0.001 |
| 1P-2P-5P-3P-4N-7P-8P-10N | 0.001 | 0.212 | 0 | 0 | 0.074 | 0.002 | 0.45 | 0 | 0 |
| 1P-5P-2N-4N-8P-9P | 0.476 | 0.141 | 0 | 0 | 0.158 | 0.47 | 0 | 0.175 | 0.025 |
| 1P-5P-2N-4N-7P-8P-9P-10N | 0.008 | 0.085 | 0 | 0 | 0.025 | 0.538 | 0.053 | 0.089 | 0.003 |
| 1P-2P-5P-4N-8P-9N-10N | 0.001 | 0.585 | 0 | 0.001 | 0.12 | 0 | 0.175 | 0 | 0 |
| 1P-5P-3N-8P | 0.133 | 0.451 | 0 | 0 | 0.161 | 0.54 | 0 | 0.173 | 0.013 |
| 1P-5P-2N-3P-4N-7P-9P-10N | 0.001 | 0.02 | 0.001 | 0.001 | 0.311 | 0.53 | 0.035 | 0.079 | 0.001 |
| 1P-2P-3N-4N-8P-9P-10N | 0.003 | 0.511 | 0 | 0 | 0.079 | 0.037 | 0.371 | 0.002 | 0.003 |
| 1P-2P-3N-8P | 0.175 | 0.501 | 0 | 0.006 | 0.127 | 0.373 | 0 | 0.258 | 0.042 |
| 1P-2N-3P-4N-8P | 0.904 | 0.003 | 0 | 0.017 | 0.025 | 0.453 | 0 | 0.264 | 0.042 |
| 1P-2P-5P-3N-4N-7P-8P | 0.023 | 0.073 | 0.003 | 0.004 | 0.017 | 0.627 | 0.001 | 0.237 | 0.037 |
| 1P-5P-3N-7P | 0.014 | 0.001 | 0.009 | 0 | 0.123 | 0.663 | 0 | 0.202 | 0.007 |
| 1P-5P-2N-3P-4N-7P-8P-9P-10N | 0.008 | 0.093 | 0 | 0.01 | 0.033 | 0.535 | 0.046 | 0.087 | 0.001 |
| 1P-5P-2N-3P-7P | 0.375 | 0 | 0.002 | 0.034 | 0.132 | 0.537 | 0 | 0.157 | 0.011 |
| 1P-2P-5P-3N-4N-7P-8P-9N | 0.019 | 0.076 | 0.001 | 0.003 | 0.019 | 0.049 | 0.049 | 0.003 | 0.074 |
| 1P-2P-3P-4N-8P-10N | 0.001 | 0.608 | 0 | 0.001 | 0.115 | 0.001 | 0.412 | 0 | 0 |
| 1P-5P-3N-4N-8P-10N | 0.002 | 0.562 | 0 | 0 | 0.131 | 0.4 | 0 | 0.067 | 0 |
| 1P-5P-2N-3P-4N | 0 | 0 | 0 | 0 | 1 | 0.361 | 0.001 | 0.135 | 0.005 |
| 1P-2P-3N-7P-8P | 0.035 | 0.066 | 0.095 | 0 | 0.013 | 0.537 | 0 | 0.279 | 0.023 |
| 1P-2P-5P-3P-4N-7P-9N-10N | 0 | 0.005 | 0.005 | 0.007 | 0.484 | 0.002 | 0.223 | 0 | 0.002 |
| 1P-5P-2N-7N-8P | 0.491 | 0.195 | 0 | 0 | 0.104 | 0.219 | 0 | 0.076 | 0.013 |
| 1P-5P-2N-4N-7P-9P-10N | 0 | 0.027 | 0.002 | 0 | 0.196 | 0.555 | 0.04 | 0.089 | 0.001 |
| 1P-2P-5P-8P-10N | 0.002 | 0.561 | 0 | 0.007 | 0.123 | 0.034 | 0.187 | 0 | 0.001 |
| 1P-2P-5P-4N-7P-8P-9P-10N | 0.001 | 0.269 | 0 | 0.001 | 0.069 | 0 | 0.424 | 0 | 0 |
| 1P-5P-2N | 0 | 0 | 0 | 0 | 1 | 0.389 | 0 | 0.149 | 0.015 |
| 1P-5P-2N-4N-7P | 0.024 | 0.015 | 0.018 | 0 | 0.501 | 0.424 | 0.01 | 0.146 | 0.016 |
| 1P-5P-2N-7P-10N | 0 | 0.002 | 0.005 | 0 | 0.127 | 0.649 | 0.008 | 0.026 | 0 |
| 1P-2P-5P-3N-4N-7P-8P-10N | 0 | 0.099 | 0.002 | 0.004 | 0.014 | 0.57 | 0.051 | 0.109 | 0.003 |
| 1P-5P-2N-3N-4N-7P-8P | 0.086 | 0.059 | 0.002 | 0 | 0.026 | 0.566 | 0.003 | 0.137 | 0.029 |
| 1P-2P-5P-3N-4N-9N | 0 | 0 | 0 | 0 | 1 | 0.087 | 0.029 | 0.013 | 0.057 |
| 1P-2P-5P-3P-4N-7P-10N | 0.001 | 0 | 0.003 | 0.002 | 0.505 | 0.002 | 0.441 | 0.001 | 0.001 |
| 1P-2P-5P-4N-7P-8P-9N-10N | 0 | 0.243 | 0.001 | 0.001 | 0.058 | 0 | 0.207 | 0 | 0 |
| 1P-5P-2N-4N-9P | 0 | 0.003 | 0 | 0 | 0.978 | 0.475 | 0.005 | 0.148 | 0.015 |
| 1P-5P-3P-4N-8P-10N | 0 | 0.604 | 0 | 0.001 | 0.101 | 0.013 | 0.097 | 0 | 0 |
| 1P-5P-3P-7P-10N | 0 | 0.002 | 0.001 | 0.007 | 0.135 | 0.411 | 0.05 | 0.008 | 0.001 |
| 1P-5P-2N-3P-4N-9P-10N | 0 | 0.003 | 0 | 0 | 0.723 | 0.48 | 0.029 | 0.068 | 0.001 |
| 1P-5P-3P-8P-10N | 0.001 | 0.568 | 0 | 0.007 | 0.124 | 0.049 | 0.096 | 0 | 0 |
| 1P-2P-3N-4N-7P-8P-10N | 0.001 | 0.284 | 0.035 | 0 | 0.065 | 0.039 | 0.377 | 0.001 | 0.013 |
| 1P-2P-3N-4N-10N | 0 | 0 | 0 | 0 | 1 | 0.03 | 0.172 | 0.096 | 0.011 |
| 1P-2P-3P-4N-9P-10N | 0 | 0 | 0.007 | 0 | 0.846 | 0.002 | 0.363 | 0.001 | 0 |
| 1P-2P-3P-4N-8P-9N-10N | 0.001 | 0.656 | 0 | 0 | 0.095 | 0.001 | 0.199 | 0 | 0 |
| 1P-2P-5P-4N-7P-9P-10N | 0 | 0.002 | 0.012 | 0 | 0.529 | 0.001 | 0.389 | 0 | 0 |
| 1P-5P-2N-4N-7P-10N | 0 | 0.013 | 0.008 | 0 | 0.195 | 0.449 | 0.044 | 0.077 | 0.001 |
| 1P-2P-4N-7P-8P-9N-10N | 0 | 0.268 | 0.035 | 0 | 0.047 | 0.001 | 0.263 | 0 | 0 |
| 1P-5P-2N-3N-4N-10N | 0 | 0 | 0 | 0 | 1 | 0.531 | 0.017 | 0.062 | 0.001 |
| 1P-5P-2N-3P-7P-8P-10N | 0.001 | 0.072 | 0.005 | 0.042 | 0.015 | 0.607 | 0.022 | 0.038 | 0 |
| 1P-2P-5P-4N-10N | 0 | 0 | 0 | 0 | 1 | 0.001 | 0.351 | 0.001 | 0 |
| 1P-2P-5P-4N-9P-10N | 0 | 0 | 0.003 | 0 | 0.81 | 0.002 | 0.35 | 0 | 0 |
| 1P-5P-3N-4N-7P-8P | 0.019 | 0.074 | 0.003 | 0 | 0.021 | 0.527 | 0.001 | 0.201 | 0.008 |
| 1P-5P-2N-3P-4N-7P-8P-9P | 0.622 | 0.005 | 0 | 0.006 | 0.149 | 0.534 | 0 | 0.175 | 0.014 |
| 1P-2P-5P-3N-4N-8P-9P-10N | 0.002 | 0.465 | 0 | 0 | 0.091 | 0.031 | 0.357 | 0.003 | 0.001 |
| 1P-2P-4N-8P-9P-10N | 0 | 0.524 | 0 | 0 | 0.082 | 0.002 | 0.477 | 0 | 0 |
| 1P-5P-2N-3N-8P | 0.173 | 0.407 | 0 | 0 | 0.156 | 0.534 | 0 | 0.075 | 0.004 |
| 1P-2P-5P-4N-7P-9N-10N | 0 | 0.004 | 0.024 | 0 | 0.432 | 0 | 0.2 | 0 | 0 |
| 1P-2P-5P-3N-4N-8P | 0.126 | 0.473 | 0 | 0.005 | 0.15 | 0.577 | 0.001 | 0.206 | 0.049 |
| 1P-5P-7P-8P-10N | 0 | 0.081 | 0 | 0 | 0.02 | 0.413 | 0.005 | 0.022 | 0 |
| 1P-5P-2N-3P-4N-8P-9P | 0.604 | 0.011 | 0 | 0.009 | 0.152 | 0.481 | 0 | 0.162 | 0.015 |
| 1P-2P-5P-3N-8P | 0.213 | 0.379 | 0 | 0.004 | 0.145 | 0.562 | 0 | 0.178 | 0.026 |
| 1P-5P-2N-3P-7P-10N | 0.002 | 0.002 | 0.014 | 0.046 | 0.128 | 0.631 | 0.012 | 0.035 | 0.001 |
| 1P-2P-5P-3N-4N-7P-8P-9N-10N | 0 | 0.117 | 0 | 0.002 | 0.013 | 0.04 | 0.075 | 0.001 | 0.014 |
| 1P-5P-7P-10N | 0 | 0.002 | 0.007 | 0 | 0.127 | 0.458 | 0.001 | 0.019 | 0 |
| 1P-5P-3N-7P-10N | 0 | 0.002 | 0.006 | 0 | 0.127 | 0.698 | 0 | 0.091 | 0 |
| 1P-2P-5P-3P-4N-9P-10N | 0 | 0 | 0 | 0 | 0.828 | 0.002 | 0.367 | 0 | 0 |
| 1P-2P-5P-3N-7P-10N | 0 | 0.002 | 0.009 | 0 | 0.127 | 0.693 | 0 | 0.111 | 0.001 |
| 1P-5P-2N-3P-8P-10N | 0.007 | 0.508 | 0 | 0.029 | 0.114 | 0.401 | 0.032 | 0.067 | 0.003 |
| 1P-2P-4N-8P-9N-10N | 0 | 0.645 | 0 | 0 | 0.1 | 0 | 0.227 | 0 | 0 |
| 1P-5P-2N-3N-4N-7N-8P | 0.231 | 0.431 | 0 | 0 | 0.107 | 0.313 | 0 | 0.061 | 0.009 |
| 1P-5P-3N-4N-7P | 0.002 | 0.002 | 0.01 | 0 | 0.127 | 0.51 | 0.001 | 0.201 | 0.001 |
| 1P-2P-5P-3P-4N-8P-9N-10N | 0.001 | 0.601 | 0 | 0 | 0.115 | 0.001 | 0.184 | 0 | 0.001 |
| 1P-5P-3N-4N-7P-9P | 0.002 | 0.002 | 0.002 | 0 | 0.099 | 0.468 | 0 | 0.168 | 0.001 |

Only robust topologies with *Q* ≥ 0.1 for at least one S2 and the sum of frequencies ≥ 0.1 are shown.
