# Supplementary material for: An Atlas of Network Topologies Reveals Design Principles for Caenorhabditis elegans Vulval Precursor Cell Fate Patterning
Source: PLoS One. 2015 Jun 26;10(6):e0131397. doi: 10.1371/journal.pone.0131397 (PMC4482679; doi:10.1371/journal.pone.0131397)

**S1 Text. The constraint of parameter sets for 1P-2P-3N to function using morphogen gradient dependent strategy.**

Analysis of parameter sets for 1P-2P-3N showed particular constraints on the parameters of the regulation from the AC to the 1° and 2° nodes. The figure below shows that the topology only functions in the conditions of high values of the parameter *k* for 1P (*k_1_*) and low values of *k* for 2P (*k_2_*). Note that low values of *k* in the Hill model indicate that the regulation is sufficient with a low signal, while high values of *k* need a high signal to execute activation or inhibition. Thus, the low values of *k* for 2P ensure that the low signal from the AC to 2° fate cells is sufficient to induce the 2° fate, but the high values of *k* for 1P make the low signal insufficient to generate the 1° fate. While in 1° cell, although both 1° and 2° fates are induced first, the induced 1° fate will repress 2° fate finally via 3N.


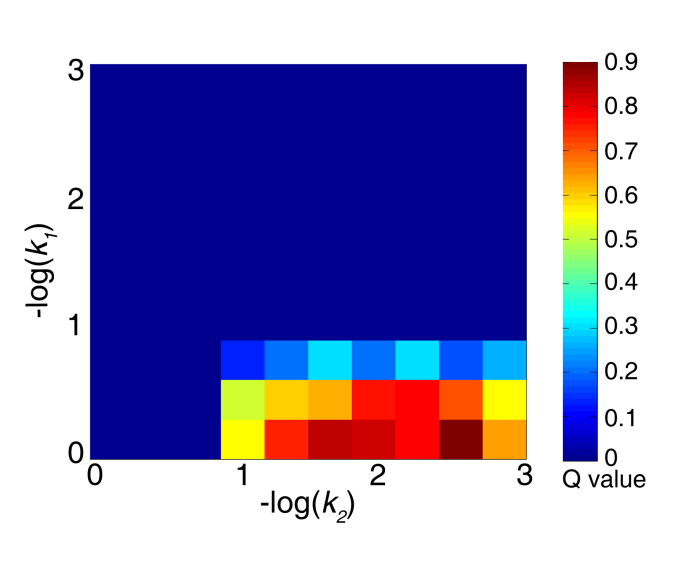

Supplement: S1 Text — (DOCX) [file pone.0131397.s019.docx]
